# Kinetic proofreading through the multi-step activation of the ZAP70 kinase underlies early T cell ligand discrimination

**DOI:** 10.1038/s41590-022-01288-x

**Published:** 2022-08-31

**Authors:** Guillaume Voisinne, Marie Locard-Paulet, Carine Froment, Emilie Maturin, Marisa Goncalves Menoita, Laura Girard, Valentin Mellado, Odile Burlet-Schiltz, Bernard Malissen, Anne Gonzalez de Peredo, Romain Roncagalli

**Affiliations:** 1 Centre d’Immunologie de Marseille-Luminy, Aix Marseille Université, INSERM, CNRS, Marseille, France; 2 Département Biologie Structural Biophysique, Institut de Pharmacologie et de Biologie Structurale, Protéomique Génopole Toulouse Midi Pyrénées CNRS UMR, Toulouse, France; 3 Centre d’Immunophénomique, Aix Marseille Université, INSERM, CNRS, Marseille, France; 4 Present Address: Novo Nordisk Foundation Center for Protein Research, University of Copenhagen, Copenhagen, Denmark

**Keywords:** T-cell receptor, Kinases

## Abstract

T cells recognize a few high-affinity antigens among a vast array of lower affinity antigens. According to the kinetic proofreading model, antigen discrimination properties could be explained by the gradual amplification of small differences in binding affinities as the signal is transduced downstream of the T cell receptor. Which early molecular events are affected by ligand affinity, and how, has not been fully resolved. Here, we used time-resolved high-throughput proteomic analyses to identify and quantify the phosphorylation events and protein–protein interactions encoding T cell ligand discrimination in antigen-experienced T cells. Although low-affinity ligands induced phosphorylation of the Cd3 chains of the T cell receptor and the interaction of Cd3 with the Zap70 kinase as strongly as high-affinity ligands, they failed to activate Zap70 to the same extent. As a result, formation of the signalosome of the Lat adaptor was severely impaired with low- compared with high-affinity ligands, whereas formation of the signalosome of the Cd6 receptor was affected only partially. Overall, this study provides a comprehensive map of molecular events associated with T cell ligand discrimination.

## Abstract (web summary)

Roncagalli and colleagues use time-resolved high-throughput proteomic analyses to provide a comprehensive picture of the impact of ligand affinity on early T cell receptor signaling.

## Main

T cells have the ability to sense and discriminate a wide range of antigenic peptides bound to major histocompatibility complex (pMHC) molecules according to their affinity for the T cell receptor (TCR). Small differences in binding kinetics between TCR ligands lead to marked differences in T cell effector function and differentiation. In particular, the magnitude of T cell responses scales with the affinity of the peptide encountered^1–4^. The kinetic proofreading model postulates that TCR ligand discrimination is due to several signaling steps that introduce a delay between ligand binding and T cell activation. This implies that the pMHC ligand dwell time on the TCR determines the probability of completing a series of signaling events leading to T cell activation^5^. Insightful studies have identified molecular mechanisms in favor of this model during T cell development or in mature T cells^6–8^. Optogenetic approaches have suggested that discrimination occurs rapidly downstream of the recruitment of the Zap70 kinase to the TCR complex^9,10^. This is consistent with reports that the slow phosphorylation of tyrosine residues of the Lat adaptor associates with recruitment and activation of the phospholipase Plcγ1, thereby constituting an important kinetic bottleneck for ligand discrimination^11^. Yet, characterization of the emergence of differences in signaling events triggered by ligands with different affinities along the TCR signaling cascade remains incomplete. Hence, how ligand affinity converts into signaling potency (that is, magnitude of T cell responses) remains unresolved. In particular, it is unclear whether ligand affinity uniformly affects the different signaling pathways involved in T cell activation.

Here, we used mass spectrometry (MS)-based methods to determine and quantify, in a time-dependent manner, early molecular events triggered following TCR stimulation with peptides of varying affinities. We defined quantitative signatures of ligand affinity based on protein phosphorylation and protein–protein interaction (PPI) stoichiometry for critical molecular events associated with TCR signaling. Our analysis indicated that ligand affinity differentially affected signaling pathways associated with specific kinases and biological processes. The divergence between signals induced by ligands with different affinities occurred early in the canonical TCR signaling cascade. While the phosphorylation of tyrosine residues in the immunoreceptor tyrosine-based activation motifs (ITAMs) of Cd3 and the inducible interaction between Cd3 and Zap70 remained largely unaffected, the abundance of activated Zap70 dropped with decreasing peptide affinity. Consequently, the phosphorylation and formation of the Lat signalosome was severely impaired under low-strength TCR stimulation. In contrast, the assembly of the Cd6 signalosome with that of the Slp76 adaptor, also mediated by Zap70 in a TCR-inducible manner, was affected only partially by ligand affinity. Together, our results suggest that ligand discrimination is encoded within the multi-step activation of the proximal tyrosine kinase Zap70.

## Results

### Ligand affinity controls the scale of T cell responses

**Fig. 1:**
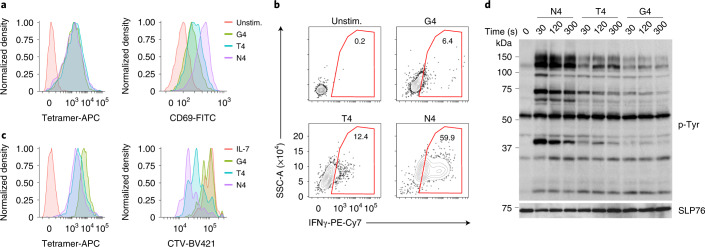
Responses of OT-I CD8^+^ T cells after stimulation with pMHC tetramers of different affinity under equal TCR occupancy conditions. **a**, Quantification of bound tetramers and CD69 expression by flow cytometry in OT-I CD8^+^ T cells purified from lymph nodes and spleen, briefly expanded in vitro with coated CD3 plus soluble CD28 antibodies for 48 h, followed by incubation with IL-2 for an additional period of 48 h before stimulation with 20 nM N4, 50 nM T4 or 300 nM G4 for 12 h. Unstim, no pMHC tetramers. **b**, Quantification of IFN-γ production by intracellular staining and flow cytometry 12 h poststimulation with tetramers and in the presence of soluble anti-CD28 as in **a**. **c**, Quantification by flow cytometry of bound tetramers and cell proliferation as measured by CTV dilution 72 h poststimulation with tetramers and in the presence of soluble anti-CD28 as described in **a**. ‘IL-7’, unstimulated control corresponding to a dose of 5 ng ml^–1^ IL-7 to maintain cell viability in the absence of tetramers. **d**, Immunoblot analysis of total protein lysates from OT-I cells left unstimulated (0 s) or stimulated with N4, T4 or G4 as described in **a** for 30–300 s. Total cellular lysates were then probed with a phosphotyrosine antibody (p-Tyr). Anti-SLP76 immunoblot served as a loading control. Molecular weights are indicated on the left. Data are representative of more than three independent experiments. Source data

To define the impact of ligand affinity on early TCR signaling events, we used mouse OT-I T cells, which specifically recognize the ovalbumin OVA_257–264_ peptide (SIINFEKL, also named N4) bound to the MHC-I molecule H-2K^b^ (pMHC). The OT-I TCR also recognizes altered peptide ligands, amongst which SIITFEKL (T4) and SIIGFEKL (G4) have suboptimal binding capacities (ligand affinity N4 > T4 > G4). To stimulate a large number of OT-I cells in a synchronous fashion, we used soluble pMHC tetramers laden with N4, T4 or G4 peptides (hereafter N4, T4 and G4) (Fig. 1). For a given tetramer concentration, the average number of TCR bound at equilibrium scales with ligand affinity and these differences in receptor occupancy propagate to downstream signaling events^12^. To correct for this effect, we selected different concentrations of N4, T4 and G4 that induced equal TCR occupancy (Fig. 1a). As expected, N4 stimulation led to efficient T cell expansion, upregulation of Cd69 and cytokine secretion (Fig. 1a–c). Consistently, CD8^+^ OT-I T cells stimulated with N4 for 30, 120 or 300 s triggered an increase of global tyrosine phosphorylation compared with the unstimulated condition (Fig. 1d). Stimulation with T4 or G4 with similar TCR occupancy showed reduced T cell proliferation, cytokine secretion and tyrosine phosphorylation events compared with N4 stimulation conditions (Fig. 1a–d). Hence, when a similar number of TCR were engaged, ligand affinity correlated with stimulation potency as defined by the magnitude of proximal and distal T cell activation readouts.

### Ligand affinity defines the set of phosphorylation events

**Fig. 2:**
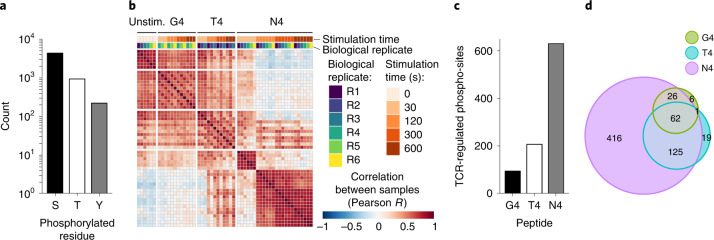
Global analysis of T cell phosphoproteome as a function of TCR ligand affinity. **a**, Number of phosphorylated serines (S), threonines (T) and tyrosines (Y) identified in the phosphoproteome of OT-1 CD8^+^T cells after stimulation with N4, T4 or G4 (Extended Data Fig. 1). **b**, Heatmap displaying the correlation (Pearson) of phosphopeptide intensity between each biological replicates (R1–R6) and stimulatory conditions. Unstim, no pMHC tetramers. **c**, Number of phospho-sites significantly regulated upon TCR stimulation with G4, T4 or N4. **d**, Euler diagram showing the repartition of the regulated phospho-sites between stimulations with G4, T4 or N4.

**Extended Data Fig. 1:**
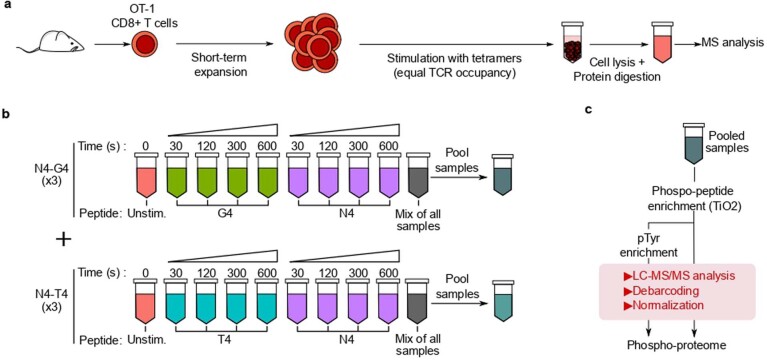
Workflow for the analysis of T cell phosphoproteome as a function of TCR ligand affinity. (**a**) Experimental workflow to quantify phosphorylations occurring in the 10 min following TCR stimulation with N4, T4 or G4 under equal TCR occupancy conditions as described in Fig. 1a. OT-1 T cells were stimulated with N4, T4 or G4 for 30, 120, 300, or 600 seconds before lysis, protein digestion and MS analysis. Control samples were harvested without stimulation (0 s). (**b**) Schematics of the multiplexing strategy employed to compare phosphorylation intensities across a large set of samples. Peptides from different stimulatory condition were labelled with isobaric tags (TMT) enabling multiplexed MS analysis. Six independent experiments consisting of three replicates with N4 and T4 stimulations (N4-T4) and three replicates with N4 and G4 stimulations (N4-G4) were conducted and analyzed to generate the phosphoproteomic dataset. (**c**) Strategy for phospho-peptide enrichment and MS analysis (see the Methods section for more details).

To capture the molecular events driving ligand potency on a broad scale and in a hypothesis-free fashion, we first determined the phosphoproteome of briefly expanded CD8^+^ OT-I T cells left unstimulated or stimulated with N4, T4 or G4 for 30, 120, 300 or 600 s (Extended Data Fig. 1a). To detect subtle differences between experimental conditions, we used a multiplexing method based on mass-isobaric labelling (referred to as tandem mass tag (TMT)) that reduces intersample variability (Extended Data Fig. 1b; Methods). We conducted six independent experiments, each involving either stimulations with N4 and T4 or with N4 and G4 (Extended Data Fig. 1b). This resulted in six biological replicates for the unstimulated condition and N4 stimulations, which were used for interexperiment normalization, and three biological replicates for T4 and G4 stimulations. For each experiment, barcoded samples were pooled and subjected to phospho-enrichment using TiO_2_ beads. Considering that phosphorylation on tyrosine residues (pY) accounts for only 1–2% of cellular phosphorylation events, we enriched pY using immunoprecipitation (IP) (pY-IP) on the TiO_2_ eluates^13^ (Extended Data Fig. 1c). We quantified 5,699 phospho-sites corresponding to 5,532 unique individual phosphorylated residues on 1,851 proteins (Fig. 2a and Supplementary Table 1). Among them, we identified 4,365 sites on serine residues (pS), 943 on threonine residues (pT) and 224 on tyrosine residues (pY). To obtain a general overview of this phospho-dataset, we computed correlations between samples corresponding to different experimental conditions and biological replicates. This showed a strong correlation between replicates from the same stimulatory condition (Fig. 2b), confirming the good reproducibility between experiments. It also highlighted differences between unstimulated samples and samples stimulated with pMHC tetramers bound to low (G4)-, medium (T4)- or high (N4)-affinity peptides, indicating that differences between ligand affinities can be identified by global phosphoproteomics.

**Extended Data Fig. 2:**
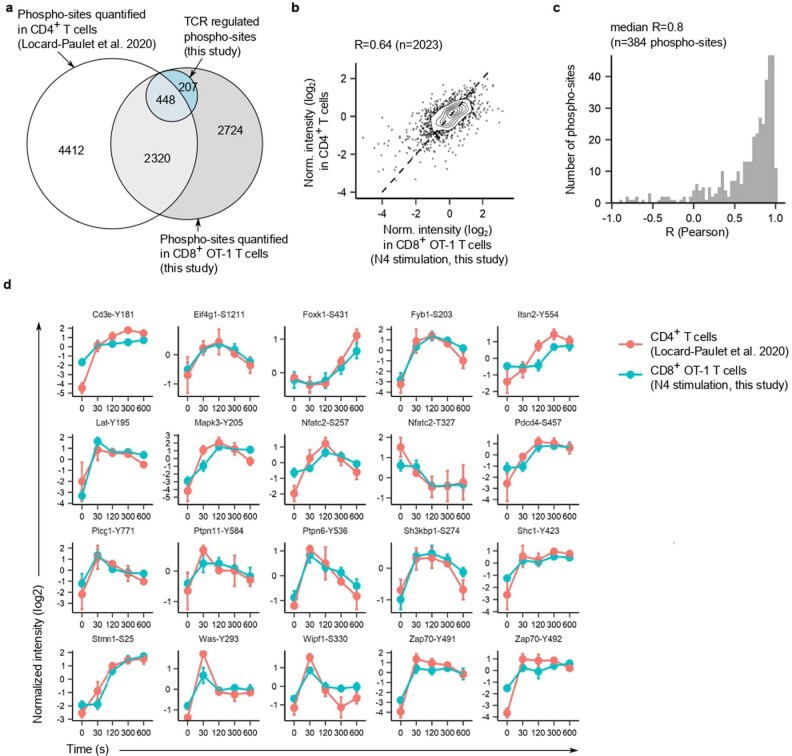
Comparative analysis of the phosphoproteomes of OT-I CD8^+^ and CD4^+^ T cells. (**a**) Euler diagram representing the repartition of phospho-sites identified and regulated in the present study relative to those identified in our previous study using CD4^+^ T cells (Locard-Paulet et al. 2020). (**b**) Comparison of phospho-site intensities (normalized across biological replicates) between CD4^+^ T cells stimulated with anti-CD4 and anti-CD3 antibodies and OT-I CD8^+^ T cells stimulated with N4. Only phospho-sites identified as regulated upon TCR stimulation in both studies were considered. Pearson correlation coefficient R = 0.64. (**c**) Distribution of Pearson correlation coefficients comparing log-transformed normalized phosphosite intensities between N4-stimulated OT-1 CD8^+^ T cells (this study) and antibody stimulated CD4^+^ T cells (Locard-Paulet et al. 2020) across phospho-sites identified as regulated upon TCR stimulation in both studies. Phospho-site intensities were normalized as in (b). The correlation coefficient was computed only for phospho-sites with intensity values available in both datasets for a minimum of four stimulatory conditions (n = 384 phospho-sites). **(d)** Overlay dynamics of selected phospho-sites in N4-stimulated OT-1 CD8^+^ T cells (this study) and antibody-stimulated CD4^+^ T cells^13^. Error bars represent standard deviation across biological replicates (n = 6 for OT-1 CD8^+^ T cells, n = 4 for CD4^+^ T cells).

To determine which phospho-sites were regulated upon TCR stimulation, we applied a statistical analysis workflow^13^ in which phospho-sites were considered regulated when they presented well-resolved kinetics, with an absolute fold change of at least 1.5 associated with a corrected *P* value ≤ 0.05 between two time points (Methods). We identified 655 phospho-sites (or combination of sites) significantly regulated in the course of TCR stimulation with either N4, T4 or G4 (Supplementary Table 1). The phosphorylation kinetics of the TCR-regulated phospho-sites after N4 stimulation were similar to those obtained upon stimulation of CD4^+^ T cells with CD3ε + CD4 antibodies^13^ (Extended Data Fig. 2). This analysis provided an extensive picture of TCR-regulated molecular events affecting proximal TCR signaling components (Cd3 chains, Zap70, Lck), signal integrators (Plcγ1, Lat, Vav1) and distal effectors (Erk1/2, Akt2, Nfatc2) (Supplementary Table 1). Among the 655 regulated phospho-sites, 629, 207 and 95 were triggered by N4, T4 or G4 stimulation, respectively (Fig. 2c). The vast majority of phospho-sites significantly regulated by G4 (92%) and T4 (90%) were also regulated by N4 (Fig. 2d). In addition, most of the G4-regulated phospho-sites (66%) were also regulated by T4. Hence, the set of regulated phospho-sites expands gradually with increasing ligand affinity.

### Ligand affinity modulates specific signaling pathways

**Fig. 3:**
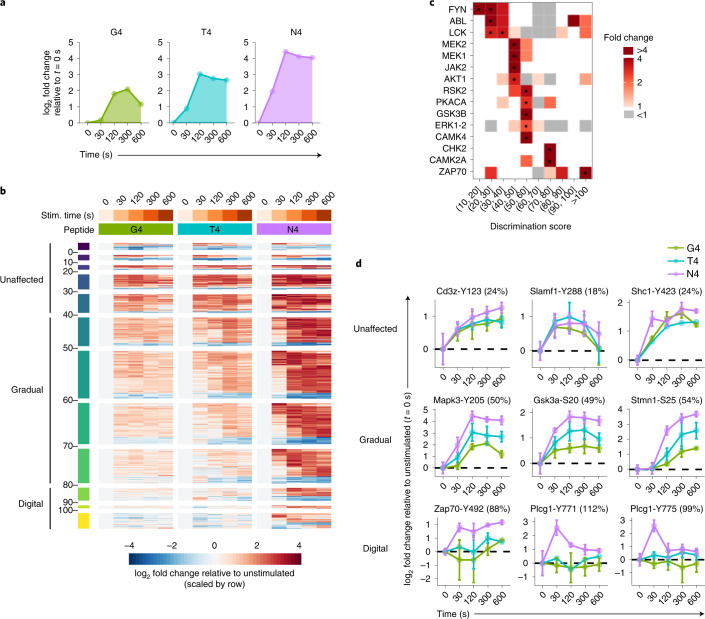
Analysis of phosphorylation regulation as a function of TCR ligand affinity. **a**, Illustration of phosphorylation kinetics for G4, T4 and N4 stimulations. The areas under the curve for the responses to G4, T4 or N4 stimulations shown here are equal to 5.2, 9.3 and 14.6, respectively. This corresponds to response losses of 64% for G4 and 36% for T4 as compared with N4, which yield a discrimination score (average loss) equal to 50% in this scenario. **b**, Heatmap displaying the log_2_ fold change between stimulated and unstimulated conditions for all TCR-regulated phospho-sites for which phosphorylation could be quantified across all experimental conditions. For each phospho-site (row), the log_2_ fold change was scaled by dividing by the s.d. Phospho-sites were ranked according to the discrimination score calculated as described in **a** and their kinetics of phosphorylation. Three main response profiles were defined based on the discrimination score: Unaffected (discrimination score ≤40%), Gradual (discrimination score >40%, and ≤80%) and Digital (discrimination score >80%). **c**, Heatmap showing kinase-specific substrate enrichment as a function of the discrimination score (*x* axis). Black dots indicate statistically significant enrichment (hypergeometric test *P* value ≤ 0.05, fold change ≥2, number of substrates ≥2; gray squares, fold change <1). **d**, Representative activation profiles of phosphorylation sites presenting Unaffected, Gradual or Digital responses as described in **b**. Log_2_-transformed fold changes relative to the average intensity in unstimulated controls (*t* = 0 s) are plotted for each time point. Error bars represent s.d. across biological replicates (*n* = 6 for N4 and unstimulated, *n* = 3 for T4 and G4). Discrimination scores are reported in parenthesis in the graph titles.

To examine how the magnitude of phosphorylation responses varied as a function of ligand affinity, we selected 452 TCR-regulated phospho-sites for which the phosphorylation intensity could be quantified across all peptides and stimulation times. We calculated fold changes of phosphorylation intensities between stimulated and unstimulated conditions and defined a discrimination score comparing the responses of the altered peptides (T4, G4) with that of the agonist peptide (N4) (Fig. 3a). The discrimination score quantified the average percentage of decrease in response resulting from T4 and G4 stimulations as compared with N4 stimulation across all stimulation times: 0% indicated events unaltered by change in ligand affinity, whereas 100% indicated that only the N4 stimulation triggered a response. According to the discrimination score, the phospho-sites were partitioned into 12 groups (Fig. 3b). Phospho-sites with a low discrimination score (below 40%) displayed an amplitude of phosphorylation largely independent of TCR-peptide affinity and were qualified as unaffected (Fig. 3b). A second type of response was qualified as gradual (discrimination score between 40% and 80%) and corresponded to phospho-sites with a response amplitude scaling with ligand affinity (Fig. 3b). Finally, a third group of phospho-sites that qualified as digital (discrimination score above 80%) were specifically regulated upon N4 stimulation, but unresponsive to stimulation with weaker affinity peptides (Fig. 3b).

**Extended Data Fig. 3:**
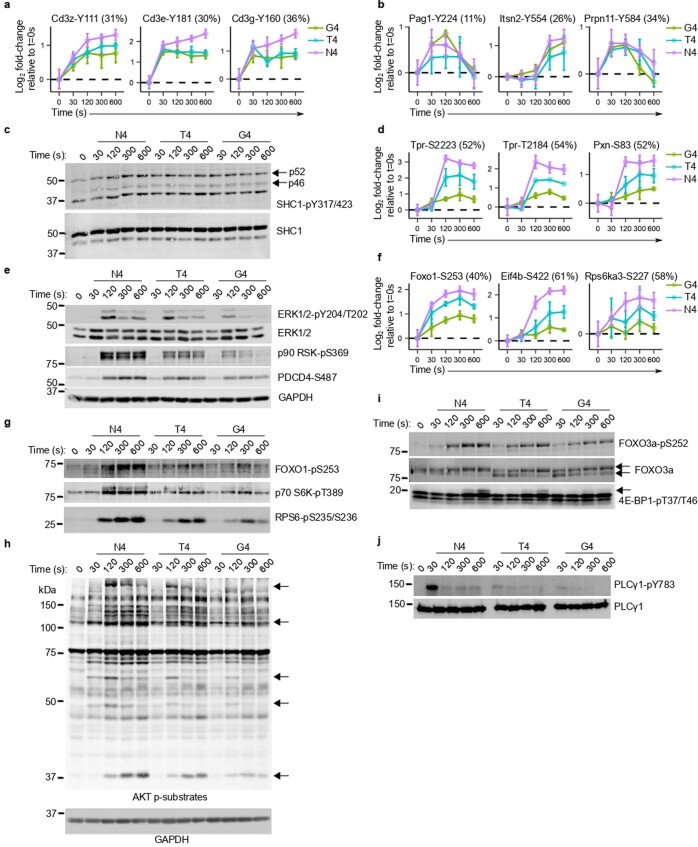
Impact of TCR ligand affinity on TCR signaling effectors. Analysis of phosphorylation intensity between stimulated and unstimulated conditions for selected phospho-sites associated with unaffected (**a**-**c**), gradual (**d**-**i**) or digital responses (**j**). (**a,b**) Representation of log_2_-transformed fold-changes of the phosphorylation intensity relative to the average intensity in unstimulated controls (t = 0 s) for selected phospho-sites identified in the phospho-dataset. Error bars represent standard deviation across biological replicates (n = 6 for N4 and unstimulated, n = 3 for T4 and G4). Discrimination scores are reported in parenthesis. (**c**) Immunoblot analysis of total protein lysates from OT-I cells left unstimulated (t = 0 s) or stimulated with N4, T4 or G4 under equal TCR occupancy conditions as described in Fig. 1a. T cells were stimulated for the indicated times and probed with the indicated phospho-specific antibodies. Arrows in the margins indicate mass shifts due to protein isoforms or phospho-isoforms of interest. Molecular weights are indicated on the left. (**d**) Analysis of phosphorylation intensity between stimulated and unstimulated conditions for selected phospho-sites associated with gradual responses displayed as in (a). (**e**) Immunoblot of total protein lysates displaying gradual responses analyzed as in (c). (**f**) Analysis of phosphorylation intensity between stimulated and unstimulated conditions for selected phospho-sites associated with gradual responses displayed as in (a). (**g**-**j**) Immunoblot of total protein lysates displaying gradual (g-i) or digital (j) responses analyzed as in (c). Data are representative of three independent experiments. Source data

To determine whether the unaffected, gradual or digital responses reflected specific kinase activities, we mapped kinase–substrate relationships from the PhosphoSitePlus database (phosphosite.org) and analyzed substrate enrichment according to the discrimination score. The unaffected sites were enriched for substrates of the SRC kinases Fyn and Lck (Fig. 3c). Among them, the presence of pY of the Cd3 chains (Cd3ζ-Y83/Y111/Y123/Y142/Y153, Cd3ε-Y181 and Cd3δ-Y149) indicated that, under equal TCR occupancy, phosphorylation of ITAMs was independent of ligand affinity (Fig. 3d and Extended Data Fig. 3a). Other phospho-sites subjected to the regulation of SRC kinases and localized in the intracytoplasmic domains of SLAM family receptors (Slamf1-Y288, Slamf6-Y319 and Slamf3-Y625) and of the Pag1 transmembrane molecule (Pag1-Y224) were also independent of peptide affinity (Fig. 3d, Extended Data Fig. 3b and Supplementary Table 1). The unaffected category also contained intracytoplasmic effectors with inhibitory functions such as Ptpn11 (also Shp2; Y546, Y584), Itsn2 (Y922, Y554) and Shc1 (Y423) (Fig. 3d and Extended Data Fig. 3b,c), suggesting that some negative signals were equally induced by both low- and high-affinity ligands^13–16^. Therefore, some SRC kinase signals were similar between T cells stimulated with weak and strong agonists.

Substrates of kinases involved in the Mapk and Akt-mTOR signaling pathways were enriched in the set of phospho-sites displaying gradual responses (Fig. 3c). Accordingly, the amplitude of Erk1/Erk2 phosphorylation and its known downstream substrates such as Tpr, Stmn1 and Pxn scaled with peptide affinity (Fig. 3d and Extended Data Fig. 3d). This type of response was confirmed by the immunoblot analyses of p-Erk1/2 and its previously identified substrates^13^, Rsk1 (S369) and Pdcd4 (S457)—the latter being coincident with translation initiation (Extended Data Fig. 3e and Supplementary Table 1). Such a TCR-affinity-dependent response was also associated with the phosphorylation of classical substrates from the Akt-mTOR signaling pathway, such as Gsk3a/b, Eif4b and Foxo1 (Fig. 3d and Extended Data Fig. 3f,g). These observations were supported by the phosphorylation pattern of immunoblots probed with an Akt phospho-substrate antibody and extended to additional known Akt-mTOR substrates that were not detected in this phospho-dataset, such as Foxo3a (S253) and Eif4ebp1 (T37/46) (Extended Data Fig. 3h,i). Finally, substrates that can be phosphorylated either by Mapk^13^ and Mtorc1 (refs. ^17,18^), such as Rps6kb1 and Rps6, also showed a gradual phosphorylation intensity as a function of peptide affinity (Extended Data Fig. 3g).

Phospho-sites presenting a digital response were significantly enriched for some of Zap70 regulatory sites and for known substrates of this kinase (Fig. 3c), including Plcγ1 pY sites (Fig. 3d and Extended Data Fig. 3j), indicating that Zap70 phosphorylation and activity depend greatly on ligand affinity. Altogether, the analysis of phosphorylation responses as a function of ligand affinity revealed distinct patterns associated with different canonical signaling pathways.

### Ligand affinity regulates stoichiometry of protein interactions

**Fig. 4:**
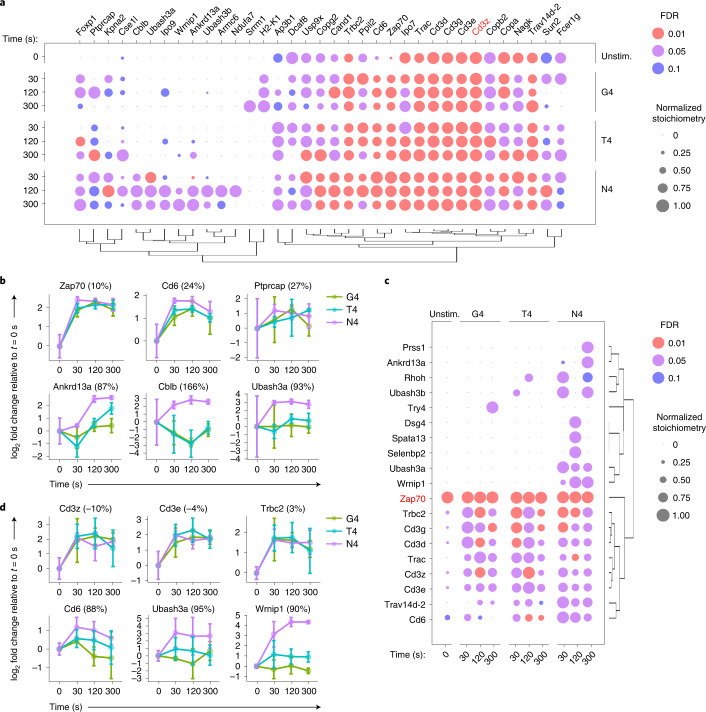
Analysis of the Cd3ζ and Zap70 signalosomes as a function of TCR ligand affinity. **a**, Dot plot showing the time-resolved interaction stoichiometry of proteins significantly associated with Cd3ζ^OST^ in unstimulated (Umstim.) or stimulated conditions. T cells were stimulated with N4, T4 or G4 under equal TCR occupancy conditions as described in Fig. 1a. Dots are colored-coded according to the enrichment FDR value and only conditions with FDR values below 0.1 are represented. Proteins were considered significantly associated when presenting an enrichment FDR ≤ 0.05 and a fold change ≥10 in at least one experimental condition. The interaction stoichiometry of each protein was normalized by the maximal value across all experimental conditions. Proteins were ordered by hierarchical clustering based on their row-wise normalized stoichiometry. **b**, Representation of log_2_ fold changes of stoichiometry relative to the average stoichiometry in unstimulated controls (*t* = 0 s) for selected interactors of CD3ζ^OST^ as a function of stimulation time and peptide affinity. Discrimination scores, computed as described in Fig. 3a, are reported in parenthesis. Error bars represent s.d. across biological replicates (*n* = 6 for N4 and unstimulated, *n* = 3 for T4 and G4). **c**, Dot plot showing the time-resolved interaction stoichiometry of proteins significantly associated with Zap70^OST^ analyzed as in **a**. **d**, Representation of log_2_ fold changes of stoichiometry relative to the average stoichiometry in unstimulated controls (*t* = 0 s) for selected interactors of Zap70^OST^ as a function of stimulation time and peptide affinity. Discrimination scores, computed as described in Fig. 3a, are reported in parenthesis. Error bars represent s.d. across biological replicates (*n* = 6 for N4 and unstim., *n* = 3 for T4 and G4).

**Extended Data Fig. 4:**
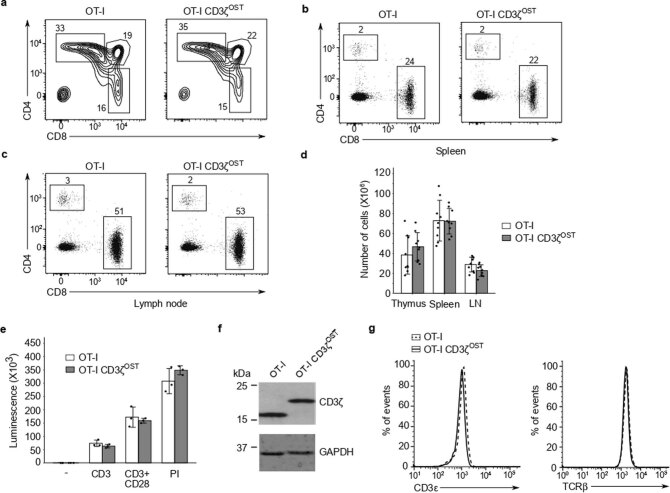
Normal development and function of T cells from the OT-I Cd3ε^OST^ mouse. (**a**-**c**) Flow cytometry analysis of thymus (a), Spleen (b) and Lymph nodes (c). Wild-type OT-I and OT-I Cd3ζ^OST^ thymocytes were analyzed for expression of CD4 and CD8. Numbers adjacent to the outlined areas indicate percent double-positive cells in (a), and CD4 + or CD8 + single-positive cells in (a) to (c). Data are representative of at least three experiments with three mice per genotype. (**d**) Cellularity of thymus, pooled axillary, brachial, inguinal and mesenteric lymph nodes (LN) and spleen of wild-type OT-I and OT-I Cd3ζ^OST^ mice. Data are expressed as mean value ± SD (n = 9 mice integrated over three independent experiments).(**e**) Proliferative capacity of wild-type OT-I and OT-I Cd3ζ^OST^ CD8^+^ T cells activated for 72 h with plate-bound anti-CD3 (3 μg/ml) in the presence or absence of soluble anti-CD28 (1 μg/ml) or with PMA/Ionomycin (PI). The ATP present in the culture medium was assessed by luminescence as a measure of the extent of cell proliferation. Data are expressed as mean value ± s.d (n = 3 mice representative of three independent experiments). (**f**) Immunoblot analysis of total protein lysates from wild-type OT-I and OT-I Cd3ζ^OST^ CD8^+^ T cells probed with anti-CD3ζ or anti-GAPDH (loading control) antibodies. Molecular weights are indicated on the left. Data are representative of three independent experiments. (**g**) Flow cytometry analysis showing expression of CD3ε and TCRβ of peripheral CD8^+^T cells from OT-I and OT-I Cd3ζ^OST^ mice. Data are representative of three independent experiments.

**Extended Data Fig. 5:**
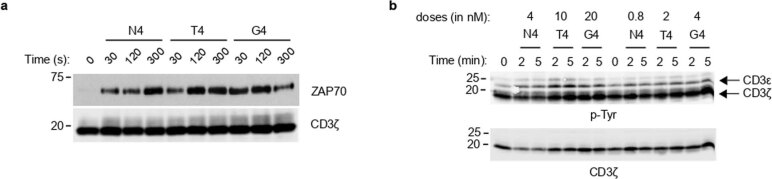
Cd3-Zap70 interaction and Cd3 phosphorylation as function of ligand affinity. Immunoblot analysis of affinity-purified Cd3ζ molecules from OT-I CD3 ζ ^OST^ cells that have been left unstimulated (t = 0 s) or stimulated with N4, T4 or G4 for the indicated times. (**a**) Immunoblot was probed with anti-ZAP70 and anti-CD3ζ. (**b**) Two different levels of occupancy have been calculated and tested according to two N4 doses (4 and 0.8 nM). Immunoblot was probed with antibodies specific for phospho-tyrosines (p-Tyr) and CD3ζ. Phospho-species of CD3 chains are annotated with arrows. Molecular weights are indicated on the left (kDa). Data are representative of three independent experiments. Source data

To quantify how ligand affinity affected protein–protein interactions involved in proximal TCR signaling, we crossed OT-I TCR transgenic mice onto mice expressing an endogenously tagged (one-strep-tag, OST) version of Zap70 (Zap70^OST^)^19^ or Cd3ζ (Cd3ζ^OST^). Similar to the Zap70^OST^ mice^19^, the OT-I Cd3ζ^OST^ mice showed normal T cell development, yielded a normal number of mature T cells and had no defect in proliferative capacity (Extended Data Fig. 4a–e). Furthermore, the expression of the tagged Cd3ζ^OST^ was similar to that of untagged Cd3ζ, and the introduction of the tag did not affect the abundance of TCR on the cell surface (Extended Data Fig. 4f,g). Affinity purification (AP) and MS analysis was then used to assess the composition and stoichiometry of the protein complexes assembled around the tagged molecule (denoted as bait). OT-I Cd3ζ^OST^ and OT-I Zap70^OST^ CD8^+^ T cells were prepared as described for the phosphoproteomic analysis above, and left unstimulated or stimulated with N4, T4 or G4 for 30, 120 or 300 s. To distinguish true protein interactions from nonspecific contaminants, we compared AP-MS results between cells expressing the tagged protein and those with the endogenous version of the same protein. The interactors that were significantly enriched over the time course of TCR stimulation (fold change >10 and false discovery rate (FDR) < 0.05 for at least one experimental condition) were displayed as a function of their interaction stoichiometry (Fig. 4 and Supplementary Table 2). Albeit present in a small amount at basal state, the recruitment of Zap70 to Cd3ζ exhibited a 5.3-fold increase following N4 stimulation for 30 s and remained persistent throughout TCR engagement (Fig. 4a,b). Conversely, a similar increase in the stoichiometry of Cd3ζ-Zap70 was observed in the Zap70 interactome upon N4 stimulation (Fig. 4c,d). To estimate the cellular abundance of these complexes, we quantified the protein copy numbers per OT-I CD8^+^ T cell by performing a global quantitative proteomic analysis by MS. The cellular abundance of Zap70-Cd3ζ complexes in OT-I CD8^+^ T cells ranged from 4.2 × 10^3^ in the unstimulated state to 22 × 10^3^ at the peak of stimulation (Supplementary Table 3). Beside the canonical Cd3ζ-Zap70 interaction, N4 stimulation also induced Cd3ζ association with other known (Cd6 or Cblb) and unreported (Ubash3a or Ubash3b) interactors, as well as with proteins that have not been yet characterized in the context of TCR signaling, such as Wrnip1 and Ankrd13a (Fig. 4a). As previously documented using pervanadate stimulation^19^, the N4-stimulated Zap70^OST^ interactome contained Cd6, the Rho-related GTP-binding protein Rhoh and the phosphatase Ubash3a (Fig. 4c). Like with Cd3ζ^OST^, we also captured Wrnip1 and Ankrd13a with Zap70^OST^ after TCR stimulation (Fig. 4c), further positioning these proteins as potential regulators of TCR signaling. As such, for both the Zap70 and Cd3ζ interactomes, the induced association between Zap70 and Cd3ζ was unaltered by peptide affinity under equal TCR occupancy conditions (Fig. 4a–d). This was further confirmed by immunoblot analysis of affinity-purified Cd3ζ ^OST^ molecules processed as in the MS analysis (Extended Data Fig. 5a). Likewise, the interaction of Cd6 with Cd3ζ occurred irrespective of peptide affinity (Fig. 4a,b)—a finding consistent with the fact that Cd6 was recruited to the same extent in the immunological synapse following stimulation with lower affinity ligands^20^. In contrast, the interaction stoichiometries of Cd6 with Zap70 scaled with peptide affinity (Fig. 4c,d). The association of Cd3ζ and/or Zap70 with Ubash3a/b, Cblb, Ankrd13a and Wrnip1 showed a strong reduction of stoichiometry, or became undetectable when T cells were stimulated with weak agonist peptides (Fig. 4a–d). In summary, the quantitative analysis of stoichiometries for protein–protein interactions participating in proximal TCR signaling indicated molecular signatures associated with stimulation strength: on the one hand the abundance of the Zap70-Cd3 complexes was unchanged, and protein complexes containing Cd6 formed even with weak-affinity peptides; on the other hand, many downstream protein–protein interactions were partially or completely lost with a ligand of medium or low affinity.

### The abundance of active Zap70 controls ligand discrimination

**Fig. 5:**
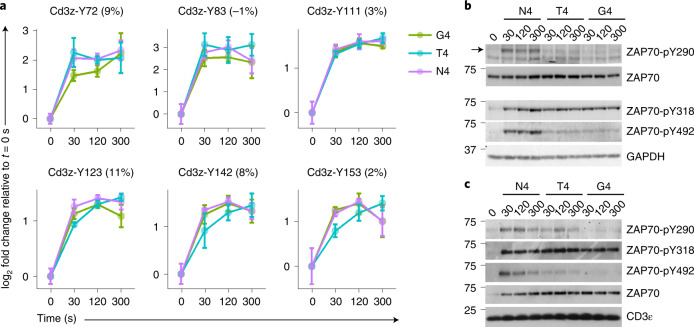
Impact of TCR ligand affinity on proximal phosphorylation events. **a**, Representation of log_2_-transformed fold changes of phosphorylation intensity relative to the average intensity in unstimulated controls (*t* = 0 s) for phosphorylation on tyrosine residues within ITAMs of the Cd3ζ^OST^ molecule. Phosphorylation intensities were quantified from affinity-purified Cd3ζ^OST^ samples. Discrimination scores, computed as described in Fig. 3a, are reported in parenthesis. Error bars represent s.d. across biological replicates (*n* = 6 for N4 and unstimulated, *n* = 3 for T4 and G4). **b**, Immunoblot analysis of total protein lysates from OT-I cells left unstimulated (*t* = 0 s) or stimulated with N4, T4 or G4 under equal TCR occupancy conditions as described in Fig. 1a. T cells were stimulated for the indicated times and probed with phospho-specific antibodies recognizing phosphotyrosine residues in Zap70. Anti-ZAP70 and anti-GAPDH immunoblots served as a loading control. **c**, Immunoblot analysis of affinity-purified Cd3ζ molecules from OT-I Cd3ζ^OST^ cells treated and probed as in **b**. Data are representative of three independent experiments. Source data

The activation of Zap70 requires a multi-step process regulated by successive molecular events^21^. The consensus model states that Zap70 can bind to doubly phosphorylated ITAMs, but that this recruitment is insufficient to induce full kinase activity^21^. The second regulatory step consists of Lck phosphorylation of critical tyrosine residues in the interdomain B of Zap70 (Y290, Y314 and Y318 in mouse), which promotes its active conformation. The ultimate event is associated with increased phosphorylation of Zap70 Y491 and Y492 likely initiated by Lck, and subsequently enhanced by transphosphorylation of Zap70 bound to the TCR complex. To assess the events that occur after TCR engagement, we quantified ITAM phosphorylation from purified Cd3 complexes and extracted the intensity of peptides containing pY located within ITAMs from the affinity-purified Cd3ζ^OST^ experiments. Analysis of the amplitude and dynamics of phosphorylation of these ITAM-located phospho-sites showed no significant differences between stimulations performed with N4, T4 or G4 (Fig. 5a). TCR-induced Cd3ε phosphorylation was also minimally affected when lower doses of peptides were used, while maintaining the same TCR occupancy (Extended Data Fig. 5b).

Because phosphorylation of the Cd3 chains and the Cd3ζ-Zap70 interaction were independent of peptide affinity, we analyzed the phospho-regulations occurring on Zap70 in more detail. As the AP-MS analysis did not monitor all Zap70 phospho-sites with sufficient confidence, we focused on tyrosines Y290, Y318 and Y492, for which specific antibodies are available. Immunoblot analysis of Zap70 phosphorylation from total lysates of OT-I CD8^+^ T cells stimulated with N4, T4 or G4 indicated distinct phosphorylation patterns depending on the pY probed (Fig. 5b). The TCR-driven phosphorylation of pY492 and pY290 was markedly impaired after stimulation with T4 and G4 (Fig. 5b), while Y318 phosphorylation was similarly induced with weak (G4)-, medium (T4)- and strong (N4)-agonist ligands (Fig. 5b). Next, we assessed the phosphorylation of Zap70 molecules that were associated specifically with the Cd3ζ chains. We purified Cd3ζ^OST^ and probed Zap70 phosphorylation with antibodies for pY290, pY318 and pY492. Although a similar amount of Zap70 was recovered following stimulation with N4, T4 or G4, the phosphorylation of Zap70-Y492 and Zap70-Y290 was markedly reduced after T4 and G4 stimulations as compared with the N4 stimulation (Fig. 5c). Akin to the phosphorylation of Zap70 probed in total-cell lysate, Zap70-Y318 phosphorylation on CD3ζ-associated Zap70 was minimally affected between N4, T4 or G4 stimulations (Fig. 5c). Altogether, these results indicate that neither the phosphorylation of the Cd3 chains nor the stoichiometry of the Zap70-Cd3 interaction could explain differences in ligand potency. Rather, ligand discrimination seemed to arise from differences in the phosphorylation of Zap70 on tyrosine residues that were associated with its kinase activity.

### Ligand affinity controls TCR-signal diversification

**Fig. 6:**
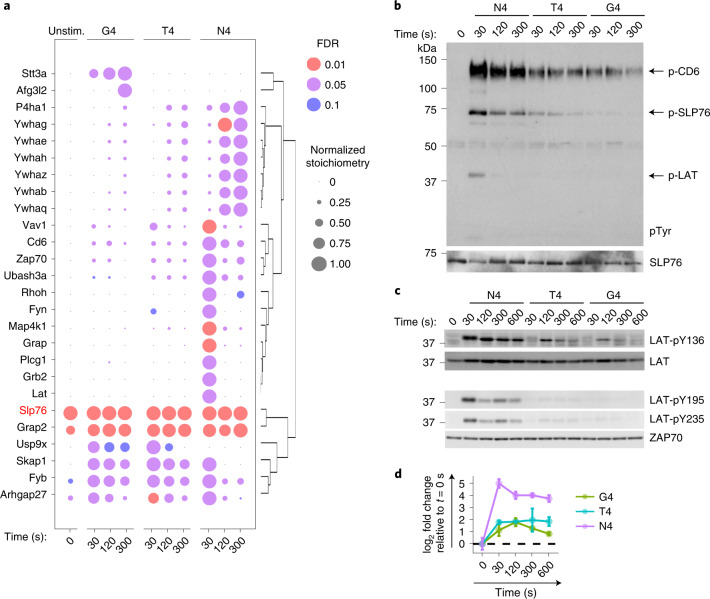
Analysis of the Slp76 signalosome as a function of TCR ligand affinity. **a**, Dot plot showing the time-resolved interaction stoichiometry of proteins significantly associated with Slp76^OST^ in unstimulated (Unstim.) or stimulated conditions. T cells were stimulated with N4, T4 or G4 under equal TCR occupancy conditions as described in Fig. 1a. Dots are colored-coded according to the enrichment FDR value, and only conditions with FDR values below 0.1 are represented. Proteins were considered significantly associated when presenting an enrichment FDR ≤ 0.05 and a fold change ≥10 in at least one experimental condition. The interaction stoichiometry of each interacting protein was normalized by the maximal value across all experimental conditions. Proteins were ordered by hierarchical clustering based on their row-wise normalized stoichiometry. **b**, Immunoblot analysis of affinity-purified Slp76 molecules from OT-I Slp76^OST^ cells that have been left unstimulated (*t* = 0 s) or stimulated with N4, T4 or G4 as described in Fig. 1a for the indicated times and probed with antibody to phosphorylated tyrosine (p-Tyr) or anti-SLP76. Phospho-molecules corresponding to Slp76, Lat and Cd6 are indicated by arrows. **c**, Immunoblot analysis of total protein lysates from OT-I cells left unstimulated (*t* = 0 s) or stimulated with N4, T4 or G4 as described in Fig. 1a for the indicated times and probed with a phospho-specific antibody recognizing the phosphorylated residues Y136, Y195 and Y235 in Lat. Data in **b** and **c** are representative of three independent experiments. Molecular weights are indicated on the left. **d**, Representation of log_2_-transformed fold changes of the phosphorylation intensity relative to the average intensity in unstimulated controls (*t* = 0 s) for the phosphorylation of the Y195 residue of Lat in the global phosphoproteome dataset. Error bars represent s.d. across biological replicates (*n* = 6 for N4 and unstimulated, *n* = 3 for T4 and G4). Source data

We next examined the molecular events immediately downstream of Zap70 activation. To probe the composition of the molecular complex that formed around Slp76, a direct substrate of Zap70, as a function of ligand affinity, we performed AP-MS on OT-I CD8^+^ T cells from mice expressing an endogenous tagged version of the Slp76 molecule (Slp76^OST^)^19,22^. Stimulation with N4 induced a robust assembly of the Slp76 signalosome (Fig. 6a and Supplementary Table 2). The Slp76 signalosome included the transmembrane proteins Lat and Cd6, as well as their associated interaction partners, such as Plcγ1 and Map4k1 (also called Hpk1) for Lat and Ubash3a for Cd6 (refs. ^19,23^). Proteins recruited to Slp76 also included effectors that can associate with both the Lat and the Cd6 signalosomes, such as the Grb2-family adaptors (Grap2, Grap and Grb2) and the guanine-exchange factor (GEF) Vav1. Zap70 was also enriched significantly with Slp76 upon stimulation (Fig. 6a). Finally, late interactions (from 120 s to 300 s) were characterized by the recruitment of the six 14–3–3 chaperones (YWHAs) (Fig. 6a), leading to the dismantling of the Slp76 signalosomes. Therefore, the N4-induced Slp76 interactome was in agreement with those deduced from stimulation with pervanadate or CD3ε antibodies^19,22^.

When the OT-I CD8^+^ T cells were stimulated with medium (T4)- or low (G4)-affinity ligands, the stoichiometries of the PPIs characterizing the Slp76 interactome were markedly changed compared with N4 (Fig. 6a and Supplementary Table 2). The Lat-specific signalosome complex was unable to assemble with Slp76, while the interactions of Slp76 with Cd6 and Ubash3a, which are the hallmarks of the second signalosome^23^, exhibited a stoichiometry scaling with peptide potency (at time *t* = 30 s, 28% and 32% of the Slp76-Cd6 interactions present under N4 stimulation were also present under G4 and T4 stimulations, respectively) (Fig. 6a). Other components of the Slp76 signalosomes (Vav1 and YWHAs) also displayed a reduced stoichiometry of interaction after T4 and G4 stimulations as compared with the N4 stimulation (Fig. 6a), presumably reflecting their capacity to participate in the Slp76-Cd6 signalosome under weaker TCR stimulation.

The assembly of the Cd6 signalosome is dependent on Zap70 activity^19^. Consistently, the stoichiometry of the interaction between Slp76 and Zap70 followed a similar trend across the different stimulation conditions to that of the interaction between Slp76 and Cd6 (Fig. 6a). Compared with N4 stimulation, immunoblot analysis performed on affinity-purified Slp76^OST^ complexes showed a reduced, but induced and persistent, phosphorylation of Cd6 after stimulation with T4 and G4 (Fig. 6b). In contrast, phospho-Lat remained undetectable after stimulation with T4 and G4 (Fig. 6b). The same strong decrease in phosphorylation was observed by immunoblotting the Lat residues Y136, Y195 and Y235 (Fig. 6c). This was in agreement with the strong decrease of Lat phosphorylation on residue Y195 under stimulation with weak (T4 and G4)-agonist ligands extracted from the phospho-dataset (Fig. 6d). These findings showed that the Slp76-Lat signalosome assembly was severely compromised, while the Slp76-Cd6 signalosome formed, albeit to a lesser extent, following stimulation with peptide of low-to-medium affinity.

### Cd6 regulates negative signals induced by weak-affinity ligands

**Fig. 7:**
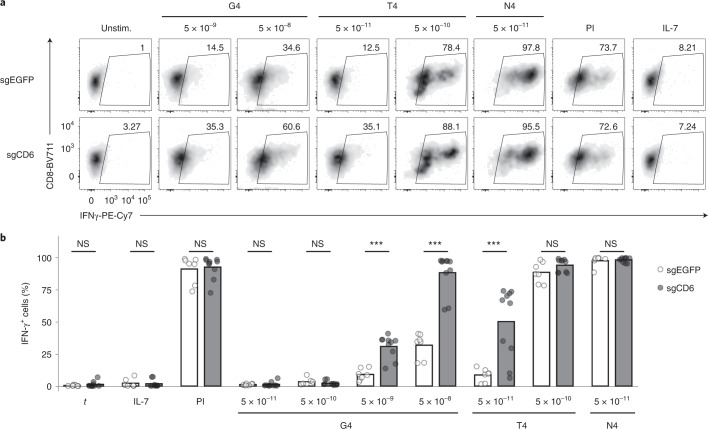
Deletion of CD6 enhances T cell responses to low-affinity TCR ligands. **a**, Flow cytometry analysis of IFN-γ-producing ability in OT-I CD8^+^T cells from Cas9-expressing mice nucleofected with sgRNA targeting Cd6 or EGFP, left to rest for 3 days and subsequently restimulated with the indicated concentration of N4, T4 or G4 for 24 h. IL-7 and PMA/ionomycin (PI) treatments were used as negative and positive controls respectively. Data are representative of three independent experiments. **b**, Bar graph depicting the percentages of IFN-γ^+^ cells after stimulation with the indicated concentrations of G4, T4, N4 or with IL-7 and PI as in **a**. (Unpaired two-tailed Welch *t*-test with *n* = 7 and *n* = 10 independent nucleofections of sgEGFP and sgCD6 respectively; NS, not significant; *P ≤ 0.05, **P ≤ 0.01, ***P ≤ 0.001). The figure integrates data from three independent experiments.

**Extended Data Fig. 6:**
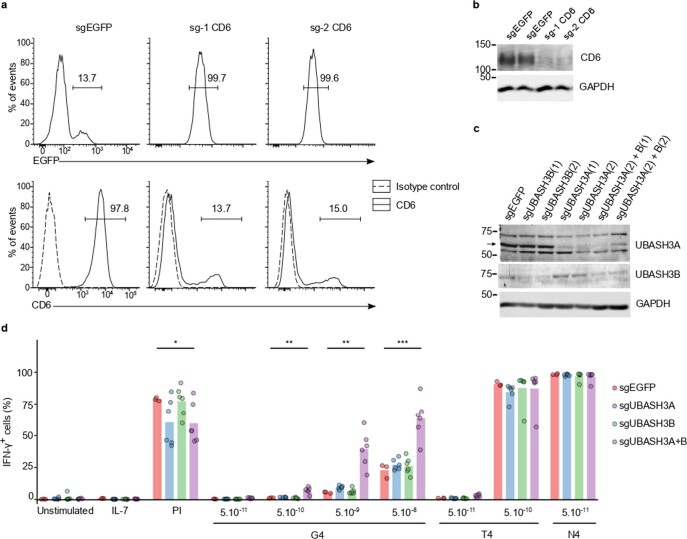
Deletion of Cd6 and Ubash3a molecules in OT-I CD8^+^ T cells. (**a**,**b**) Flow cytometry (a) and immunoblot (b) analysis of Cd6 expression in OT-I CD8^+^ T cells purified from mice constitutively expressing Cas9 and nucleofected with sgRNA targeting Cd6 or EGFP. Two different sgRNAs were used for Cd6 (sg1-CD6 and sg2-CD6). The percentage of CD6 positive cells is indicated in (a). GAPDH serves as a loading control in (b). (**c**) Immunoblot analysis of Ubash3 molecules in OT-I CD8^+^ T cells purified from mice constitutively expressing Cas9 and nucleofected with sgRNA targeting Ubash3 molecules or EGFP. Deletion efficiency of Ubash3a and Ubash3b was assessed by western blot using antibodies specific for each Ubash3 molecules. GAPDH was probed as loading control. Two different guides were used to target Ubash3a and Ubash3b molecules and are indicated in parenthesis. (**d**) Production of IFN-γ by Ubash3s targeted cells. IL-7 and PMA/ionomycin (PI) treatments were used as negative and positive controls respectively. Bar graph depicts the mean percentages of IFN-γ + cells after stimulation with the indicated concentrations of G4, T4, N4 or with IL-7 and PI. Each condition of stimulation is the results of three independent transfections of two different guides targeting specifically each Ubash3 molecules. For the double inactivation of the Ubash3 molecules, sgUBASH3A(2) has been combined with sgUBASH3B(1) or sgUBASH3B(2). (Unpaired two-tailed welch t-test with n = 6, ns: not significant; * P ≤ 0.05, ** P ≤ 0.01; *** P ≤ 0.001. Only tests for which the mean difference is greater than 5% and the p-value is lower than 5% are shown). Source data

Through its ability to recruit negative (Ubash3a) and positive (Slp76, Vav1) effectors, Cd6 is believed to generate opposing signals upon TCR stimulation^23,24^. Given the differential impact of peptide affinity on Cd6-signalosome assembly, we evaluated the contribution of Cd6 in T cell responses to weak agonist stimulation using a CRISPR–Cas9 editing system to inactivate the *Cd6* gene in OT-I CD8^+^ T cells and subsequently assess their capacity to secrete interferon (IFN)-γ^23^ (Extended Data Fig. 6a,b). Stimulation with G4 showed enhanced secretion of IFN-γ in Cd6-deficient OT-I CD8^+^ T cells compared with control OT-I CD8^+^ T cells transduced with the control guide sgEGFP (Fig. 7a,b). Although less pronounced, this difference between Cd6-deficient and wild-type (WT) OT-I CD8^+^ T cells was also observed upon stimulation with T4 (Fig. 7a,b). We also observed that OT-I CD8^+^ T cells in which both Ubash3 molecules were simultaneously inactivated using a similar approach, showed enhanced secretion of IFN-γ compared with control sgEGFP OT-I CD8^+^ T cells when stimulated with G4 (Extended Data Fig. 6c,d). These results suggested that, under conditions of weak agonist stimulation, the negative signal mediated by Cd6 was dominant, and possibly contributed to the generation of a negative feedback loop dampening T cell responses.

## Discussion

Here, using phosphoproteomics and interactomics approaches, we show that peptide affinity dictates qualitatively and quantitatively the phosphorylation and activation of molecular components controlling TCR signaling, as well as the assembly of protein complexes involved in that pathway. We found that while the very first signaling steps were unchanged upon stimulation with antigens of different affinity, low affinity antigens were unable to activate the catalytic residues of Zap70, then leading to marked defects in assembly of the TCR signalosome.

The number of ITAMs and the chronological order in which they are phosphorylated upon TCR engagement has been postulated to be one of the mechanisms encoding ligand discrimination^25,26^. We did not observe significant differences in ITAMs phosphorylation on Cd3ζ nor other Cd3 chains as a function of ligand affinity. This suggests that SRCs kinase activity does not depend on TCR-peptide affinity. Accordingly, other canonical SRC substrates, such as Pag1 or the Slam family receptors, were similarly phosphorylated by strong- or weak-affinity peptides, like phospho-sites on inhibitory cytosolic effectors (Ptpn11, Itsn2 and Shc1). Theoretical works suggest that a proximal negative feedback could enhance ligand discrimination efficiency^27^, with tyrosine phosphatase Ptpn6 (also Shp-1) being initially identified as a potential mediator^28^. Our data suggest that this negative feedback could be encoded by a more complex signal involving several molecular components.

The mechanisms underlying Zap70 activation have been investigated extensively^21^. A consensual finding is that Zap70-Cd3ζ interaction is not sufficient to fully induce Zap70 activation. This is in agreement with our data where Zap70-Cd3ζ association, independent of TCR ligand affinity, did not correlate fully with Zap70-driven downstream signaling. Indeed, the association of Zap70 to the phosphorylated Cd3ζ ITAMs was peptide-affinity-independent, whereas phosphorylation of specific Zap70 sites correlated with peptide potency. No notable differences were observed for Zap70-Y318 phosphorylation, suggesting that Zap70 N-terminal catalytic domain release can occur even upon stimulation with weak ligands^29–31^. Conversely, a strong phosphorylation decrease was measured on Zap70 Y491-Y492, localized in the activation loop and preferentially induced by Zap70 transphosphorylation^32^. According to the kinetic proofreading model, one explanation of this result would be that weak-affinity ligands unbind the TCR before completion of the transphosphorylation reaction. In agreement with other studies^33–35^, this could be associated with a lower degree of TCR oligomerization in the presence of weak-affinity peptides.

The TCR signaling diversification induced by Zap70 is generally attributed to its capacity to phosphorylate the Lat and Slp76 adaptors. Although weak agonists stimulation could induce the phosphorylation of some Lat molecules, it was insufficient to assemble productive Lat-Slp76 signalosomes. Full T cell activation requires the successive mobilization of the plasma membrane-resident Lat and the Lat molecules contained in intracellular vesicles^36,37^. Our observations suggested that stimulation with low-affinity peptides failed to activate all pools of Lat, preventing signaling to persist. On the other hand, even in these conditions of low-strength stimulation, we could detect the presence of a reduced but persistent signaling hub centered on Cd6. As for Zap70, recruitment of Cd6 to the TCR complex was similarly induced by stimulations with high and low affinity peptides. Partial Zap70 catalytic activity may be sufficient to promote Cd6 phosphorylation and, thus, some productive Cd6 signalosomes, which delivers positive and negative signals through Cd6 interaction with Slp76-Vav1 and Ubash3a, respectively^23,24^. Cd6 deletion in mature T cells has a minimal impact under strong TCR stimulation^23^, suggesting neutralization by opposing signaling effectors. In contrast, our data indicated that suboptimal stimulation by weak agonist peptide mediated predominantly the negative function of Cd6, presumably through the recruitment of Ubash3 molecules^38^. This suggests that weak agonists could preferentially signal through the Cd6 signalosome, rather than through Lat.

The probability of Lat condensates formation is determined by the TCR ligand dwell time. For a single TCR, the formation rate of a Lat condensate rises for 15 s after ligand engagement and declines back to zero after approximately 1 min (ref. ^39^). The initial rise is consistent with the existence of about two kinetic proofreading steps, which is close to the estimate of the T cell discrimination power deduced from the analysis of T cell responses to ligands of varying affinities^40^. Our results showing that discrimination occurred after Zap70 recruitment to the ITAMs and at the level of Zap70 activity and Lat phosphorylation support the existence of this relatively low number of discrimination steps. The latter decline likely reflected the existence of a local negative feedback established during the first minute of TCR engagement. Thus, according to the results provided by Cd6 and Ubash3a/b inactivation, it is tempting to speculate that this negative feedback is mediated by the Cd6 signalosome.

By integrating phosphoproteomic and interactomic analyses, our study provides a comprehensive picture of the impact of ligand affinity on early TCR signaling. We specifically highlighted the key roles of Zap70 and Lat in implementing TCR ligand discrimination. Besides all the molecular events described here, we quantified many other phosphorylation events and protein–protein interactions as a function of time and peptide affinity, which may benefit the efforts to better understand the molecular mechanisms encoding TCR ligand discrimination, and provide new perspectives for reprogramming T cell responses in autoimmune diseases and cancer.

## Methods

### Mice

OT-I TCR transgenic mice, Cas9-EGFP-expressing Gt (ROSA) 26Sor^tm1.1(CAG-cas9*,-EGFP) Fezh^^41^, Slp76^OST^ (B6-Lcp2^tm2Mal^), Zap70^OST^ (B6- Zap70^tm5Mal^) and Cd3ζ^OST^ (B6-Cd247^Tm1Ciphe^) mice were maintained in specific pathogen-free conditions at the Centre d’ImmunoPhénomique (agreement B1301407) or the Centre d’Immunologie de Marseille-Luminy (agreement F13005), and all experiments were done in accordance with national and international guidelines for laboratory animal welfare and experimentation (EEC Council Directive 2010/63/EU, September 2010). For all experiments and strains, mice were sex matched and of 8–10 weeks of age. Slp76^OST^ (B6-Lcp2^tm2Mal^) and Zap70^OST^ (B6- Zap70^tm5Mal^) mice are described in Roncagalli et al.^19^. For the Cd3ζ^OST^ (B6-Cd247^Tm1Ciphe^) mouse, the generic approach already described in Voisinne et al.^22^ was used to construct the targeting vector permitting to introduce a Twin-Strep-tag-coding sequence (OST^42^) at the 3′ end of Cd3ζ. Screening for the presence of the OST-targeted allele was performed by PCR using the pair of primers: 5′-GGTCTCAGCACTGCCACCAA-3′ and 5′-GGCAAGTGAGAGAACCATCC-3′. This pair of primers amplified a 196-bp band for the WT allele and a 377-bp band for the OST-targeted allele.

### Flow cytometry

Stained cells were analyzed using an LSRII system (BD Biosciences). Data were analyzed with the Diva software v.8 (BD Biosciences) and FlowJo v.10. Cell viability was evaluated using SYTOX Blue (Life Technologies). The following antibodies were used: anti-CD5 (BD Biosciences, catalog no. 550035, clone 53–7.3, DF 1:800), anti-CD4 (BD Biosciences, catalog no. 557956, clone RM4-5, DF 1:800), anti-CD8α (BD Biosciences, catalog no. 563046, clone 53–6.7, DF 1:400), anti-TCRβ (BD Biosciences, catalog no. 562839, clone H57-597, DF 1:200), anti-CD44 (BD Biosciences, catalog no. 560569, clone IM7, DF 1:800), anti-CD69 (BD Biosciences, catalog no. 553236, clone H1.2F3, DF 1:400), anti-CD6 (BD Biosciences, catalog no. 566426, clone J90-462, DF 1:800), anti-CD3ε (Biolegend, catalog no. B209683, clone 145-2C11, DF 1:200) and the anti-IFN-γ (Biolegend, catalog no. 505826, clone XMG1.2, DF 1:600).

### T cell isolation and short-term expansion

OT-I CD8^+^ T cells were purified from pooled lymph nodes and spleens with a Dynabeads Untouched Mouse CD8^+^T Cell Kit (Life Technologies); cell purity was 95%. CD8^+^ purified T cells were expanded for 48 h with plate-bound anti-CD3 (145-2C11, 5 μg ml^–1^) and soluble anti-CD28 (37-51; 1 μg ml^–1^ both from EXBIO). Then, T cells were harvested and grown in the presence of IL-2 (5–10 U ml^–1^) for 48 h before stimulation for phosphoproteomic and interactomic analysis.

### T cell proliferation and IFN-γ production

For proliferation assay, OT-I CD8^+^ T cells were stimulated with 20, 50 or 300 nM of N4, T4 or G4 tetramers, respectively (provided by the National Institutes of Health (NIH) tetramer core facility), with soluble anti-CD28 (37-51; EXBIO) antibody. After 48 h of culture, T cell proliferation was assessed by CTV dilution. For IFN-γ production, cells were stimulated with the same conditions and treated with GolgiStop (BD Biosciences) for the last 4 h. After incubation, cells were washed and stained for dead cells, permeabilized (fixation/permeabilization buffer; Cytofix/CytoPerm BD Biosciences) and stained for intracellular IFN-γ (XMG1.2; BioLegend) before analysis by flow cytometry.

### Deletion of the *Cd6* gene in primary OT-I CD8^+^ T cells

OT-I CD8^+^ T cells were purified by immunomagnetic negative selection from mice constitutively expressing Cas9 and edited as previously described^13^. The *Cd6* gene was ablated using the following sgRNAs (sg-1 Cd6: CCAAGGAAGAGCCACAUGUC, sg-2 Cd6: UCAGCAAUCCAGCGAUCCCA). The *Ubash3a/b* genes were ablated using the following sgRNAs (sgUBASH3A(1): UUUCCAGCAAGGGGCCCGUG, sgUBASH3A(2): UUUUCCAGCAAGGGGCCCGU, sgUBASH3B(1): UGCAGACUACUGUCAGUCGA sgUBASH3B(2): CUUCAUCGGGCUCUUCGUGA). T cells from Cas9-expressing mice also express EGFP and were used as control with the following sgRNA: GGGCGAGGAGCUGUUCACCG. The efficiency of gene deletion was checked by flow cytometry on day 3 after transfection. Edited cells were kept in culture in the presence of IL-2 (10 U ml^–1^) and IL-7 (5 ng ml^–1^) for 3 days after nucleofection. Cells were then restimulated with N4, T4 or G4 tetramers and IFN-γ production was assessed by intracellular fluorescence-activated cell sorting 24 h later.

### Stimulation of T cells for phosphoproteomic analysis

A total of 20 × 10^6^ short-term expanded OT-I CD8^+^ T cells were left unstimulated or stimulated at 37 °C with 20, 50 or 300 nM of N4, T4 or G4 tetramers, respectively. Stimulation was stopped after 30, 120, 300 or 600 s by snapfreezing the cells in liquid nitrogen. A total of six replicates were prepared for unstimulated and each time point of N4-stimulated samples, and three replicates for T4- and G4-stimulated samples.

### Phosphopeptide enrichment

The frozen cell pellets (final volume of 100 μl) were thawed and all steps until peptide digestion were performed on ice. Each pellet was resuspended in 303 μl of urea 8 M, Tris/HCl 100 mM pH 8 supplemented with PhosSTOP phosphatase inhibitor cocktail (catalog no. 04906845001, Roche), cOmplete ULTRA tablets, mini, EDTA-free (catalog no. 04693132001, Roche) and sodium orthovanadate (catalog no. S6508-10G, Sigma) 1 mM final concentration. Lysis was performed with a Bioruptor (position high, 15 cycles of 50 s/40 s) before centrifugation for 30 min (11,860*g*) at 4 °C. The protein quantity in the supernatant was measured using Bio-Rad DC protein assay kit 2 (catalog no. 500-0112-MSDS) before being kept at −80 °C until digestion. Each protein sample was subjected to filter-aided sample preparation^43^. Briefly, 1 mg of proteins per sample was concentrated with filter units (Sartorius Vivacon 500, 10 or 30 molecular weight cutoffs). Cysteine residues were reduced with 10 mM final of dithiothreitol for 40 min at 20 °C, alkylated with iodoacetamide at a final concentration of 50 mM for 25 min in the dark at room temperature. The urea concentration was then reduced by buffer exchange and the filter units were washed three times with 100 μl of triethylammonium bicarbonate (TEAB) 100 mM. Digestion was performed using trypsin (catalog no. V5117, Promega) 1% (trypsin/protein) overnight at 37˚C. Peptides were eluted from the filters by three successive centrifugation steps (one elution and two TEAB washes) before being dried down. Each peptide sample was resuspended in 500 μl of TEAB 100 mM and sonicated for complete resuspension. Next, 400 μg of peptides were independently labelled with 1.6 mg of a given TMT10 Label Reagent (TMT10plex Mass Tag Labeling Kit, catalog no. 90111, Thermo Scientific) for 1 h at room temperature. Free regents were then quenched using hydroxylamine 5% final and labelled peptides were combined (ten samples per mix: total of 4 mg of peptides), according to the multiplexing design depicted in Extended Data Fig. 1, resulting in six different TMT batches. For each batch, an aliquot of 5 μg was analyzed by MS for nonmodified peptides and protein relative quantification, and the remainder was further processed for phosphopeptide purification. All phosphopeptide enrichments were performed with Titansphere TiO_2_ beads (5 μm; GL Sciences. catalog no. 5020 75000) prewashed in TiO_2_ loading buffer (80% acetonitrile, 5% trifluoroacetic acid (TFA), 1 M glycolic acid). For each enrichment, the peptides were resuspended in TiO_2_ loading buffer (1 μg μl^–1^) and sonicated for 10 min before incubation with TiO_2_ beads 6:1 (w:w) beads:peptide for 15 min under agitation at room temperature. Beads were then sequentially washed with (1) loading buffer; (2) 80% acetonitrile, 1% TFA and (3) 10% acetonitrile, 0.2% TFA. Phosphopeptides were eluted twice with 50 μl of 1% ammonium hydroxide (pH 11.3), pooled and acidified with TFA. The eluates were then cleaned up using R3 resin packed in home-made microtips, equilibrated with 0.1% TFA. Samples were washed with 0.1% TFA and eluted with 0.1% TFA 80% acetonitrile before drying down under vacuum. The phospho-enriched peptides were further subjected to pY-IP using the PTMScan Phospho-Tyrosine Rabbit mAb (P-Tyr-1000) Kit (catalog no. 8803, Cell Signaling Technology, Ozyme) according to the manufacturer’s instructions. Briefly, phosphopeptides were resuspended on ice in cold IAP buffer (14 mg ml^–1^), sonicated for complete resuspension, and incubated with prewashed PTMScan beads (80 μl of P-Tyr bead slurry for 20 mg of protein starting material) for incubation overnight at 4 °C under mild agitation. Then, the beads were sequentially washed with IAP buffer (50 mM MOPS/NaOH, 10 mM Na_2_HPO_4_, 50 mM NaCl pH 7.2–7.4) and water before elution of the phosphopeptides with 0.15% TFA at room temperature for 10 min. Phosphotyrosine-containing peptides were then dried down under vacuum before MS analysis for phosphotyrosine relative quantification whereas the flow-throughs were analyzed for phosphoserine and phosphothreonine relative quantification.

### MS runs and search for phosphoproteomics

Samples were resuspended in 20 µl of 2% acetonitrile, 0.05% TFA, and analyzed by nanoliquid chromatography (LC) coupled to tandem MS, using an UltiMate 3000 system (NCS-3500RS Nano/Cap System, Thermo Scientific) coupled to an Orbitrap Fusion Tribrid (Thermo Scientific). Peptide samples (6 µl) were loaded and desalted on a C18 trap column (300 µm inner diameter × 5 mm, Thermo Scientific), then peptide separation was performed on an analytical C18 column (75 µm inner diameter × 50 cm, inhouse packed with Reprosil C18) equilibrated in 95% solvent A (5% acetonitrile, 0.2% formic acid) and 5% solvent B (80% acetonitrile, 0.2% formic acid), using a multi-step linear gradient of solvent B over a total of 240 min (total peptides and TiO_2_ enriched phosphopeptides) or 160 min (pY-IP enriched phosphopeptides). The fusion was operated in data dependent acquisition mode using a synchronous precursor scan-MS3 method, to avoid distortion of the ratio obtained from the TMT reporter signal when interfering peptides are co-isolated for MS2. Survey scans were performed in the Orbitrap mass analyzer at 120,000 resolution, selected parent ions were first isolated by the instrument’s quadrupole and MS2 scans were acquired in the linear ion trap using collision-induced dissociation (normalized collision energy = 35). For TMT reporter detection, MS3 scans were recorded in the Orbitrap at 60,000 resolution, following selection of the top ten precursor fragments by synchronous precursor scan and fragmentation by higher-energy collisional dissociation (normalized collision energy = 65), with an automatic gain control (AGC) target of 1 × 10^5^ and a maximum injection time of 120 ms. Dynamic exclusion was set at 20 or 30 s for pY- or TiO_2_-enriched samples, respectively. Inclusion lists were generated based on the phosphorylation sites regulated in a minimum of one condition from the analysis of the OT1 samples N4–T4. This led to the inclusion of 62 masses for the pY-IP and 610 for the TiO_2_ for the N4–G4 runs.

Raw files were analyzed with Proteome Discoverer v.2.2.0.388 using Mascot with the following parameters: Trypsin/P; maximum three missed cleavages; fragment mass tolerance 0.6 Da; precursor mass tolerance 10 ppm; fixed modification carbamidomethyl (C) and dynamic modifications phospho (ST) and (Y), oxidation (M), deamidated (NQ), acetyl (N-term); quantification method TMT10plex; target FDR was calculated using Percolator with strict of 0.01 and relaxed of 0.05 (based on *Q* value); ptmRS was used for phosphorylation site localization. Data were searched against *Mus musculus* entries of the UniProt KB protein database (release Swiss-Prot v.2017_01, 16,844 entries).

### Processing and statistical analysis of phosphorylation data

The statistical analysis was performed using the statistical package R (R Development Core Team, 2012; http://www.r-project.org/). Each TMT channel was corrected for mixing errors using the signal from nonphosphorylated samples. Only the phospho-sites with a localization score ≥75% in a minimum of one replicate were kept, and the phosphoserines and phosphothreonines were removed from the phosphotyrosine IP samples. Conversely, the phosphotyrosines quantified in the pY-IP were removed from the TiO_2_ dataset. Phosphosite intensities were log_2_-transformed and averaged across technical replicates. At this stage, missing values were replaced as follows: if for a given condition (time point, tetramer), a phosphorylation site was not quantified in any replicate experiment, missing values for that condition were imputed with low-intensity values drawn from a Gaussian distribution with mean and s.d. respectively equal to the 5% quantile and the s.d. of the entire dataset. Phosphorylation intensities were then normalized between experiments using the average intensity from N4-stimulated and unstimulated conditions—experimental conditions that were shared across all experiments.

For statistical analysis, phospho-sites with high numbers of missing values were not considered: for a given ligand affinity (tetramer), only phosphorylation sites that were detected in at least three conditions (time points) and presenting in each of them a minimum of two quantification values from the same independent replicate experiments, were retained. For those, an ANOVA test with Tukey correction was conducted to identify TCR-regulated phospho-sites. Phospho-sites were considered as significantly regulated if they presented a *P* value of the ANOVA ≤ 0.05 and a minimum of one couple of time points with a corrected *P* value ≤ 0.05 and an associated absolute fold change of greater than 1.5.

### Stimulation of T cells and preparation of samples for interactomic analysis

A total of 100 × 10^6^ short-term expanded OT-I CD8^+^ T cells were left unstimulated or stimulated at 37 °C with 20, 50 or 300 nM of N4, T4 or G4 tetramers respectively. Stimulation was stopped by the addition of a twice-concentrated lysis buffer (100 mM Tris, pH 7.5, 270 mM NaCl, 1 mM EDTA, 20% glycerol, 0.4% n-dodecyl-β-d-maltoside) supplemented with protease and phosphatase inhibitors. After 10 min of incubation on ice, cell lysates were centrifuged at 21,000*g* for 5 min at 4 °C. Postnuclear lysates were then used for AP. Equal amount of postnuclear lysates were incubated with Strep-Tactin Sepharose beads (IBA GmbH) for 1.5 h at 4 °C on a rotary wheel. Beads were then washed five times with 1 ml of lysis buffer in the absence of detergent and of protease and phosphatase inhibitors. Proteins were eluted from the Strep-Tactin Sepharose beads with 2.5 mM d-biotin—a ligand that binds to Strep-Tactin with a higher affinity than the OST sequence. Protein samples were processed for proteomic analysis either by short migration on SDS–polyacrylamide gel electrophoresis (one band) and in-gel trypsin digestion, or through protein capture on paramagnetic hydrophilic beads^44^ and in-solution trypsin digestion.

### MS runs and search for interactomics

Tryptic peptides were resuspended in 17 µl of 2% acetonitrile, 0.05% TFA and analyzed on an Orbitrap Q-Exactive Plus mass spectrometer (Thermo Fisher Scientific). Peptides were separated with the same chromatographic set-up as described above, with a 60-min or 105-min-long gradient of solvent B depending on the analytical series. The MS was operated in data-dependent acquisition mode with the Xcalibur software. MS survey scans were acquired with a resolution of 70,000 and an AGC target of 3 × 10^6^. The ten most intense ions were selected for fragmentation by high-energy collision-induced dissociation, and the resulting fragments were analyzed at a resolution of 17,500, using an AGC target of 1 × 10^5^ and a maximum fill time of 50 ms. Dynamic exclusion was used within 30 s to prevent repetitive selection of the same peptide.

Raw MS files were processed with MaxQuant software (v.1.5.2.8) for database search with the Andromeda search engine and quantitative analysis. Data were searched against *M.* *musculus* entries of the UniProt KB protein database (release UniProtKB/Swiss-Prot+TrEMBL 2017_01, 89,297 entries including isoforms), plus the One-Strep-tag peptide sequence, and the set of common contaminants provided by MaxQuant. Carbamidomethylation of cysteines was set as a fixed modification, whereas oxidation of methionine, protein N-terminal acetylation, and phosphorylation of serine, threonine and tyrosine were set as variable modifications. Specificity of trypsin digestion was set for cleavage after K or R, and two missed trypsin cleavage sites were allowed. The precursor mass tolerance was set to 20 ppm for the first search and 4.5 ppm for the main Andromeda database search. The mass tolerance in tandem MS mode was set to 0.5 Da. Minimum peptide length was set to seven amino acids, and minimum number of unique or razor peptides was set to one for validation. The I = L option of MaxQuant was enabled to avoid erroneous assignation of undistinguishable peptides belonging to very homologous proteins. Andromeda results were validated by the target decoy approach using a reverse database, with an FDR set at 1% at both peptide spectrum match and protein level. For label-free relative quantification of the samples, the match between runs option of MaxQuant was enabled with a match time window of 1 min, to allow cross-assignment of MS features detected in the different runs, after alignment of the runs with a time window of 20 min. Protein quantification was based on unique and razor peptides. The minimum ratio count was set to one for label-free quantification calculation, and computation of the iBAQ metric was also enabled.

### Data processing and identification of specific interactors

From the ‘proteinGroups.txt’ files generated by MaxQuant with the options described above, protein groups with negative identification scores were filtered out as well as proteins identified as contaminants. In situations where protein groups corresponded to the same gene name, protein intensities in a given sample were summed over the redundant protein groups. Protein intensities were normalized across all samples by the median intensity. Normalized intensities corresponding to different technical replicates were averaged (geometric mean) and missing values were replaced after estimating background binding from WT intensities. For each bait and each condition of stimulation, we used a two-tailed Welch *t*-test to compare normalized log-transformed protein intensities detected in OST-tagged samples across all biological replicates to WT intensities pooled from all conditions of stimulation. To avoid spurious identification of interactors due to missing value imputation, we repeated this process (missing value imputation followed by a two-tailed Welch *t*-test) ten times and estimated fold changes and *P* values as their respective average (geometric mean) across all ten tests. Log-transformed fold changes and corresponding *P* values were used to generate a volcano plot representing interactions across all conditions of stimulation. Asymmetry of this volcano plot was used to compute the interaction FDR as described previously^22,45^. Specific interactors were identified as preys showing a greater than tenfold enrichment with an FDR below 0.05 in at least one condition of stimulation.

### Analysis of the whole proteome of OT-I cells

For proteome analysis, OT-I cell pellets (five biological replicates) were incubated with 150 μl of lysis buffer containing Tris 50 mM, pH 7.5, EDTA 0.5 mM, NaCl 135 mM, SDS 1%, boiled 5 min at 95 °C and sonicated on a Bioruptor (Diagenode). Protein concentration was determined using a detergent compatible assay (DC assay, Bio-Rad) and an aliquot of 150 µg of protein extract was reduced (TCEP 10 mM) and alkylated (Chloroacetamide 40 mM) for 15 min at 45 °C, and digested with trypsin on a S-trap Mini device (Protifi) according to the manufacturer’s protocol. The resulting peptides were further fractionated (Pierce High pH Reversed-Phase Peptide Fractionation Kit, catalog no. 84868) into eight fractions. Each of them was dried, resuspended in 17 µl of 2% acetonitrile, 0.05% TFA and an aliquot (5 µl) was analyzed on an Orbitrap Q-Exactive HFX mass spectrometer (Thermo Fisher Scientific). Peptides were separated with a 60-min-long gradient of solvent B on an Acclaim PepMap C18 column (75 µm inner diameter × 50 cm, 2 µM particles, catalog no. 164942, Thermo Fisher Scientific). The MS was operated in data-dependent acquisition mode with the Xcalibur software. MS survey scans were acquired in the Orbitrap with a resolution of 60,000 and an AGC target of 3 × 10^6^. The 12 most intense ions were selected for fragmentation by high-energy collision-induced dissociation, and the resulting fragments were analyzed at a resolution of 30,000, using an AGC target of 1 × 10^5^ and a maximum fill time of 54 ms. Dynamic exclusion was used within 30 s to prevent repetitive selection of the same peptide. Raw MS files were processed with MaxQuant with the same parameters as described for affinity-purified samples, except that phosphorylation was not included as variable modification. Protein entries from the MaxQuant ‘proteinGroups.txt’ output were first filtered to eliminate entries from reverse and contaminant databases. Cellular protein abundances were determined from raw intensities using the protein ruler methodology^46^, using the following relationship: protein copies per cell = (protein MS signal × N_A_ × mDNA)/(M × histone MS signal), where N_A_ is Avogadro’s constant, M is the molar mass of the protein and mDNA is the DNA mass of a diploid mouse cell estimated to be 5.5209 pg. Cellular protein abundances were averaged (geometric mean) over biological replicates. Overall, the cellular protein abundance could be estimated for 4,988 protein groups.

### Western blot and antibodies

For biochemistry analysis, OT-I CD8^+^ T cells were stimulated for indicated times at 37 °C with 20 nM of N4, 50 nM of T4 or 300 nM of G4 tetramers. Stimulation was stopped by the addition of a twice-concentrated lysis buffer (100 mM Tris, pH 7.5, 270 mM NaCl, 1 mM EDTA, 20% glycerol, 0.4% n-dodecyl-β-maltoside) supplemented with protease and phosphatase inhibitors. After 10 min of incubation on ice, cell lysates were centrifuged at 21,000*g* for 5 min at 4 °C. Postnuclear lysates were used for whole cell lysates for subsequent immunoblot analysis. The following antibodies were used for immunoblot analysis: anti-SLP76 (Cell Signaling Technology, catalog no. 4958), anti-ZAP70 (Cell Signaling Technology, catalog no. 2705, clone 99F2), anti-ZAP70-pY318 (Cell Signaling Technology, catalog no. 2701), anti-ZAP70-pY492 (Cell Signaling Technology, catalog no. 2704), anti-4E-BP1-pT37/T45 (Cell Signaling Technology, catalog no. 2855, clone 234B4), anti-PLCγ1-pY783 (Cell Signaling Technology, catalog no. 2821), anti-PLCγ1 (Cell Signaling Technology, catalog no. 2822), anti-LAT-pY220 (Cell Signaling Technology, catalog no. 20172, clone E3S5L), anti-LAT-pY255 (Cell Signaling Technology, catalog no. 45170), anti-ERK1/2-pY204/T202 (Cell Signaling Technology, catalog no. 9106, clone E10), anti-ERK1/2 (Cell Signaling Technology, catalog no. 9102), anti-FOXO3-pS252 (Cell Signaling Technology, catalog no. 13129, clone D18H8), anti-FOXO3 (Cell Signaling Technology, catalog no. 12829, clone D19A7), anti-SHC1-pY317/423 (Cell Signaling Technology, catalog no. 2431), anti-RPS6-pS235/ 236 (Cell Signaling Technology, cat 4858, clone D57.2.2E), anti-P70S6K-pT389 (Cell Signaling Technology, catalog no. 9206, clone 9206), anti-p90RSK1-S369 (Cell Signaling Technology, catalog no. 12032, clone D5D8), phospho-AKT substrates (Cell Signaling Technology, catalog no. 9611), anti-ZAP70-Y290 (Biolegend, catalog no. 691902, clone A16038A), anti-PDCD4- pS457 (Thermo Fisher, catalog no. PA5-38806), anti-LAT-pY132 (Thermo Fisher, catalog no. 44-224), anti-CD6 from (Novus Biologicals, catalog no. MAB727, clone 96123) and global anti-pY (Millipore, catalog no. 16-105, clone 4G10). All residue numbering is based on the mouse protein sequences.

### Reporting summary

Further information on research design is available in the Nature Research Reporting Summary linked to this article.

## Online content

Any methods, additional references, Nature Research reporting summaries, source data, extended data, supplementary information, acknowledgements, peer review information; details of author contributions and competing interests; and statements of data and code availability are available at 10.1038/s41590-022-01288-x.

## Supplementary information

Reporting Summary

Supplementary Tables Supplementary Tables 1–3. Table 1: Phosphoproteome dataset of OT-I CD8+T cells. Related to Figs. 2, 3 and 6 and Extended Data Figs. 2 and 3. Table 2: Interactome dataset of OT-I CD8+T cells. Related to Figs. 4, 5 and 6. Table 3: Proteome dataset of OT-I CD8+T cells.

## Data Availability

The MS proteomic data have been deposited to the ProteomeXchange Consortium (http://proteomecentral.proteomexchange.org) via the PRIDE partner repository with the dataset identifiers PXD030080 (phosphoproteomic data) and PXD029974 (interactomics data). The kinase–substrate relationships were extracted from the PhosphoSitePlus database (phosphosite.org). Source data are provided with this paper.

## Source data

Source Data Fig. 1 Unprocessed western blots of Fig. 1.
Source Data Fig. 5 Unprocessed western blots of Fig. 5.
Source Data Fig. 6 Unprocessed western blots of Fig. 6.
Source Data Extended Data Fig. 3 Unprocessed western blots of Extended Data Fig. 3.
Source Data Extended Data Fig. 5 Unprocessed western blots of Extended Data Fig. 5.
Source Data Extended Data Fig. 6 Unprocessed western blots of Extended Data Fig. 6.

## Code availability

R scripts corresponding to this analysis are available on Zenodo with the identifier 6642257 (https://zenodo.org/record/6642257).
