# Supplementary material for: Kinetic proofreading through the multi-step activation of the ZAP70 kinase underlies early T cell ligand discrimination
Source: Nat Immunol. 2022 Aug 31;23(9):1355–64. doi: 10.1038/s41590-022-01288-x (PMC9477740; doi:10.1038/s41590-022-01288-x)
Supplement: Supplementary file 1 — Reporting Summary [file 41590_2022_1288_MOESM1_ESM.pdf]

nature portfolio

Corresponding author(s): Romain Roncagalli
Anne Gonzalez de Peredo
Bernard Malissen

Last updated by author(s): May 4, 2022

Reporting Summary

Nature Portfolio wishes to improve the reproducibility of the work that we publish. This form provides structure for consistency and transparency in reporting. For further information on Nature Portfolio policies, see our Editorial Policies and the Editorial Policy Checklist.

Statistics

For all statistical analyses, confirm that the following items are present in the figure legend, table legend, main text, or Methods section.

n/a	Confirmed	
☐	☒	The exact sample size (*n*) for each experimental group/condition, given as a discrete number and unit of measurement
☐	☒	A statement on whether measurements were taken from distinct samples or whether the same sample was measured repeatedly
☐	☒	The statistical test(s) used AND whether they are one- or two-sided<br>*Only common tests should be described solely by name; describe more complex techniques in the Methods section.*
☐	☒	A description of all covariates tested
☐	☒	A description of any assumptions or corrections, such as tests of normality and adjustment for multiple comparisons
☐	☒	A full description of the statistical parameters including central tendency (e.g. means) or other basic estimates (e.g. regression coefficient) AND variation (e.g. standard deviation) or associated estimates of uncertainty (e.g. confidence intervals)
☐	☒	For null hypothesis testing, the test statistic (e.g. *F*, *t*, *r*) with confidence intervals, effect sizes, degrees of freedom and *P* value noted<br>*Give P values as exact values whenever suitable.*
☒	☐	For Bayesian analysis, information on the choice of priors and Markov chain Monte Carlo settings
☒	☐	For hierarchical and complex designs, identification of the appropriate level for tests and full reporting of outcomes
☐	☒	Estimates of effect sizes (e.g. Cohen's *d*, Pearson's *r*), indicating how they were calculated

Our web collection on statistics for biologists contains articles on many of the points above.

Software and code

Policy information about availability of computer code

Data collection	Raw MS files were processed with MaxQuant software (version 1.5.2.8) and Proteome Discoverer 2.2.0.388.
Data analysis	The analysis scripts used for analysis of proteomic data were written in R V4.4.1 GNU General public licence https://www.r-project.org/ and R studio V1.4.1717 GNU General public licence https://www.rstudio.com/ and are available on Zenonodo with the DOI 10.5281/zenodo.6642257 (https://zenodo.org/record/6642257)<br>Flow cytometry analysis was performed with FACSDiva software v8 BD FACSDivaTM and FlowJo V10.

For manuscripts utilizing custom algorithms or software that are central to the research but not yet described in published literature, software must be made available to editors and reviewers. We strongly encourage code deposition in a community repository (e.g. GitHub). See the Nature Portfolio guidelines for submitting code & software for further information.

Data

Policy information about availability of data

All manuscripts must include a data availability statement. This statement should provide the following information, where applicable:

- Accession codes, unique identifiers, or web links for publicly available datasets
- A description of any restrictions on data availability
- For clinical datasets or third party data, please ensure that the statement adheres to our policy

The mass spectrometry proteomic data have been deposited to the ProteomeXchange Consortium (http://proteomecentral.proteomexchange.org) via the PRIDE partner repository with the data set identifiers PXD030080 (phosphoproteomic data) and PXD029974 (interactomics data). The kinase–substrate relationships were extracted from the PhosphoSitePlus database (phosphosite.org)

Field-specific reporting

Please select the one below that is the best fit for your research. If you are not sure, read the appropriate sections before making your selection.

☒ Life sciences ☐ Behavioural & social sciences ☐ Ecological, evolutionary & environmental sciences

For a reference copy of the document with all sections, see nature.com/documents/nr-reporting-summary-flat.pdf

Life sciences study design

All studies must disclose on these points even when the disclosure is negative.

Sample size	The sample sizes were chosen from past knowledge or internal pilot experiments enabling enough power to be adequate for statistical analysis. Sample sizes are indicated in figure legends or related "Methods" section. Size of samples are defined by the number of mice (Roncagalli et al 2014, Nature Immunology; Voisinne et al 2019, Nature Immunology) required to obtain sufficient amount of proteins from primary T cells in accordance with the animal ethical rules. We usually used 6 to 8 mice for each experiment related to phosphoproteomic.
Data exclusions	No data were excluded from the analysis
Replication	All experiments were performed at least three times independently and successfully reproduced. Reproducibility of the experiments and significances of the results are described in details in figure legends and in the "Methods" section.
Randomization	Not relevant for allocation of mice. Mice were selected according to their genotypes, age and sex-matched. For MS analyses, injection of samples were injected in a random order without regard to mouse genotypes or stimulation conditions.
Blinding	Blind group assignment was not applied. Since cells from mice with the same genotype must be pooled for experiments, it is not possible to proceed blindly.

Reporting for specific materials, systems and methods

We require information from authors about some types of materials, experimental systems and methods used in many studies. Here, indicate whether each material, system or method listed is relevant to your study. If you are not sure if a list item applies to your research, read the appropriate section before selecting a response.

Materials & experimental systems

n/a	Involved in the study	
☐	☒	Antibodies
☒	☐	Eukaryotic cell lines
☒	☐	Palaeontology and archaeology
☐	☒	Animals and other organisms
☒	☐	Human research participants
☒	☐	Clinical data
☒	☐	Dual use research of concern

Methods

n/a	Involved in the study	
☒	☐	ChIP-seq
☐	☒	Flow cytometry
☒	☐	MRI-based neuroimaging

Antibodies

Antibodies used

The following antibodies were used for immunoblot analysis:
anti-SLP76 (Cell Signaling Technology, cat 4958)
anti-ZAP70 (Cell Signaling Technology, cat 2705, clone 99F2)
anti-ZAP70-pY318 (Cell Signaling Technology, cat 2701)
anti-ZAP70-pY492 (Cell Signaling Technology, cat 2704)
anti-4E-BP1-pT37/T45 (Cell Signaling Technology, cat 2855, clone 234B4))
anti-PLCg1-pY783 (Cell Signaling Technology, cat 2821)
anti-PLCg1 (Cell Signaling Technology, cat 2822)
anti-LAT-pY220 (Cell Signaling Technology, cat 20172, clone E3S5L)
anti-LAT-pY255 (Cell Signaling Technology, cat 45170)
anti-ERK1/2-pY204/T202 (Cell Signaling Technology, cat 9106, clone E10)
anti-ERK1/2 (Cell Signaling Technology, cat 9102)
anti-FOXO3-pS252 (Cell Signaling Technology, cat 13129, clone D18H8)
anti-FOXO3 (Cell Signaling Technology, cat 12829, clone D19A7)
anti-SHC1-pY317/423 (Cell Signaling Technology, cat 2431)
anti-RPS6-pS235/ 236 (Cell Signaling Technology, cat 4858, clone D57.2.2E)
anti-P70S6K-pT389 (Cell Signaling Technology, cat 9206, clone 9206)

anti-p90RSK1-S369 (Cell Signaling Technology, cat 12032, clone D5D8)
phospho-AKT substrates (Cell Signaling Technology, cat 9611)
anti-ZAP70-Y290 (Biolegend, cat 691902, clone A16038A)
anti-PDCD4- pS457 (Thermo Fisher, cat PA5-38806)
anti-LAT-pY132 (Thermo Fisher, cat 44-224)
anti-CD6 from (Novus Biologicals, cat MAB727, clone 96123)
global anti-pY (Millipore, cat 16-105, clone 4G10)
The following antibodies were used for FACS analysis:
anti-CD5 (BD Biosciences, cat 550035, clone 53–7.3, DF 1:800)
anti-CD4 (BD Biosciences, cat 557956, clone RM4-5, DF 1:800)
anti-CD8a (BD Biosciences, cat 563046, clone 53–6.7, DF 1:400)
anti-TCRb (BD Biosciences, cat 562839, clone H57-597, DF 1:200)
anti-CD44 (BD Biosciences, cat 560569, clone IM7, DF 1:800)
anti- CD69 (BD Biosciences, cat 553236, clone H1.2F3, DF 1:400)
anti-CD6 (BD Biosciences, cat 566426 J90-462, DF 1:800)
anti-CD3e (Biolegend, cat B209683, clone 145-2C11, DF 1:200)
The anti-IFNg (Biolegend, cat 505826, clone XMG1.2, DF 1:600)

Validation

All antibodies used in this study are commercially available and have been validated and commonly used in the field. The manufacturers' websites provide details regarding validations and associated referenced publications.

Animals and other organisms

Policy information about studies involving animals; ARRIVE guidelines recommended for reporting animal research

Laboratory animals

OT-I TCR transgenic mice, Cas9-EGFP-expressing Gt (ROSA) 26Sortm1.1(CAG-cas9*,-EGFP) Fezh (Platt et al, 2014, Cell), SLP76OST (B6-Lcp2tm2Mal), ZAP70OST (B6- Zap70tm5Mal) and CD3eOST (B6-Cd247Tm1Ciphe) mice were maintained in specific pathogen-free conditions at the Centre d'ImmunoPhénomique (agreement B1301407) or the Centre d'Immunologie de Marseille-Luminy (agreement F13055), and all experiments were done in accordance with national and international guidelines for laboratory animal welfare and experimentation (EEC Council Directive 2010/63/EU, September 2010). All mice were housed in ventilated cages between 19-23°C with 45-65% humidity and a 12 hour dark/light cycle. For all experiments and strains, mice were sex matched and of 8 to 10 weeks of age.

Wild animals

This did not involve the use of wild animals.

Field-collected samples

This study did not involve the use of field-collected samples.

Ethics oversight

Mice were maintained in specific pathogen-free conditions and used in accordance with institutional committee and French and European guidelines for animal care.

Note that full information on the approval of the study protocol must also be provided in the manuscript.

Flow Cytometry

Plots

Confirm that:

☒ The axis labels state the marker and fluorochrome used (e.g. CD4-FITC).

☒ The axis scales are clearly visible. Include numbers along axes only for bottom left plot of group (a 'group' is an analysis of identical markers).

☒ All plots are contour plots with outliers or pseudocolor plots.

☒ A numerical value for number of cells or percentage (with statistics) is provided.

Methodology

Sample preparation

Spleens, Lymph nodes or thymus were harvested from mice. The orgasms were smashed on a 100-micron mesh filter. Collected cells were incubated with 1X Red Blood Cell Lysis Buffer (Invitrogen cat 00-4333-57) and them washed with FACS buffer.

Instrument

A LSRII system (BD Biosciences) was used for Flow cytometry acquisition.

Software

Flow cytometry analysis was performed with FACSDiva software v8 BD FACSDivaTM and FlowJo V10

Cell population abundance

OT-I CD8+ T cells were purified from pooled lymph nodes and spleens with a Dynabeads Untouched Mouse CD8+T Cell Kit (Life Technologies); cell purity assessed by FACS analysis was 95%.

Gating strategy	Gating strategy includes a gate on FSC/SSC followed by an exclusion of doublets and dead cells positive for Sytox blue or AquaDead (ThermoFisher Scientific) staining.

☒ Tick this box to confirm that a figure exemplifying the gating strategy is provided in the Supplementary Information.
