# Supplementary figures and images for: Kinetic proofreading through the multi-step activation of the ZAP70 kinase underlies early T cell ligand discrimination
Source: Nat Immunol. 2022 Aug 31;23(9):1355–64. doi: 10.1038/s41590-022-01288-x (PMC9477740; doi:10.1038/s41590-022-01288-x)

## Source Data-Figure 1

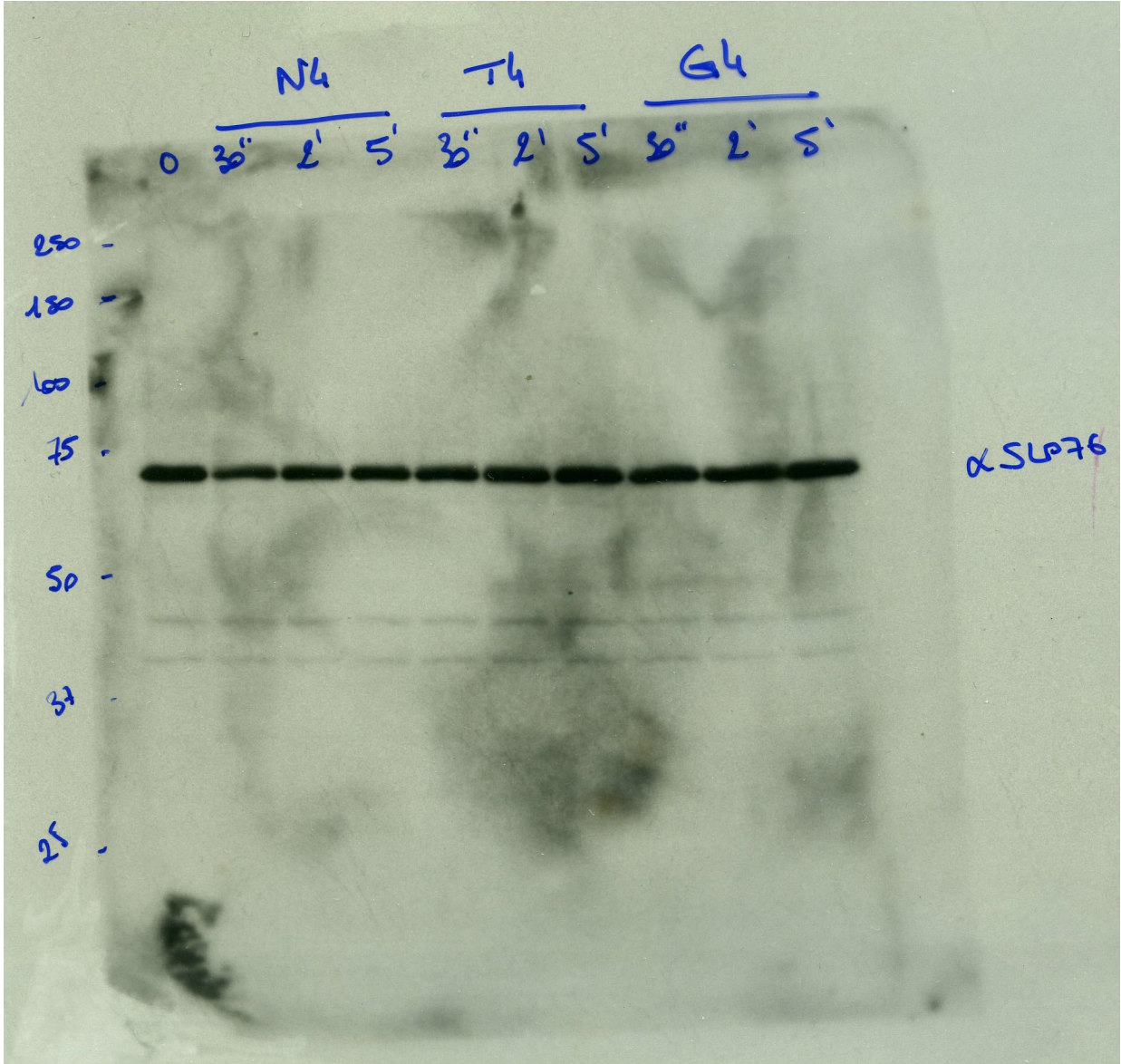

Supplement: Source Data Fig. 1 — Unprocessed western blots of Fig. 1. [file 41590_2022_1288_MOESM3_ESM.pdf]

Source Data-Figure 5b

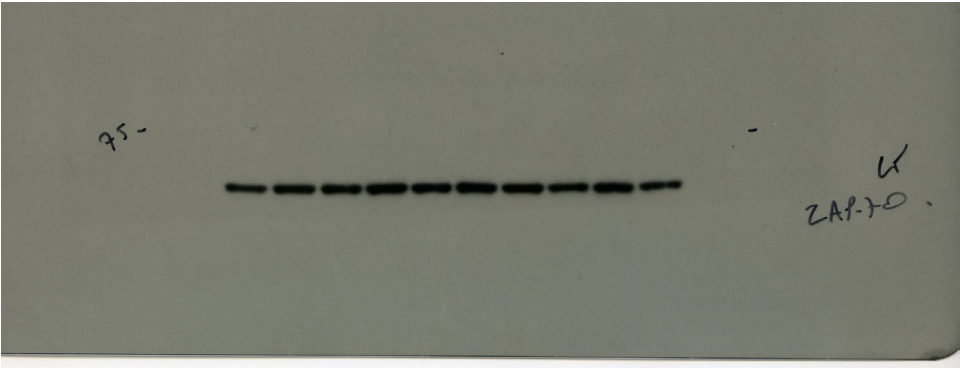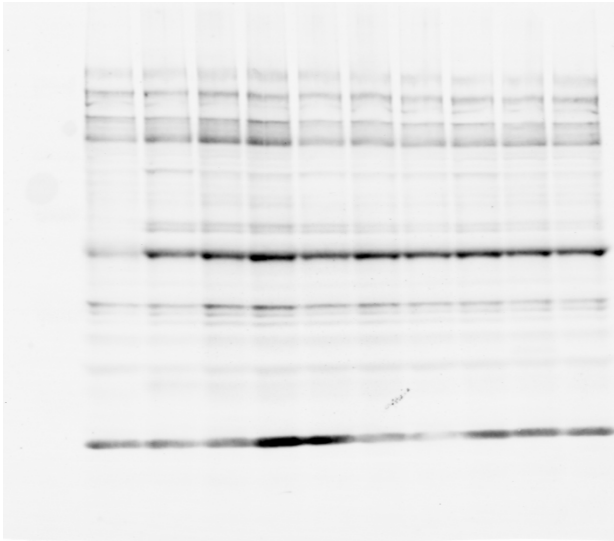

p-Y318-ZAP70

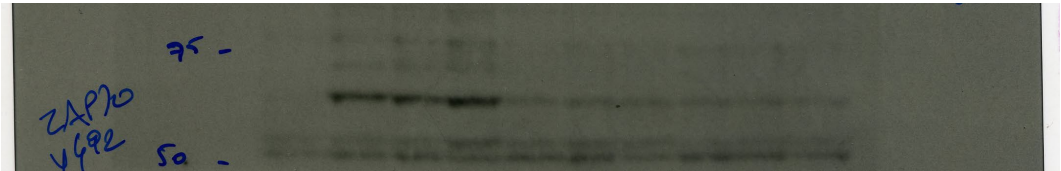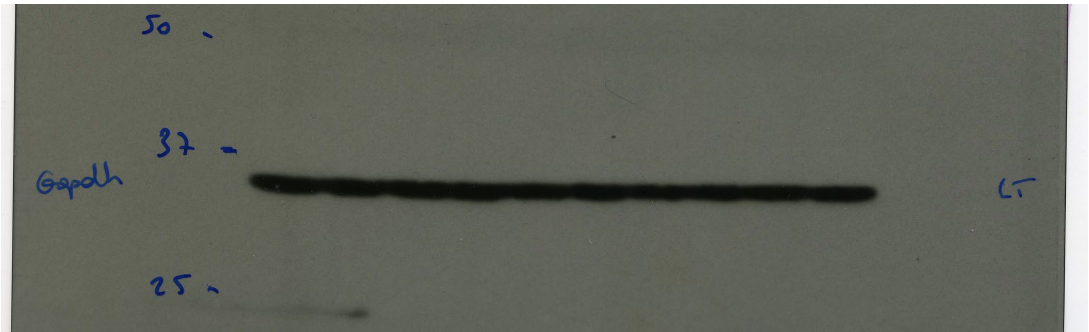

Source Data-Figure 5c

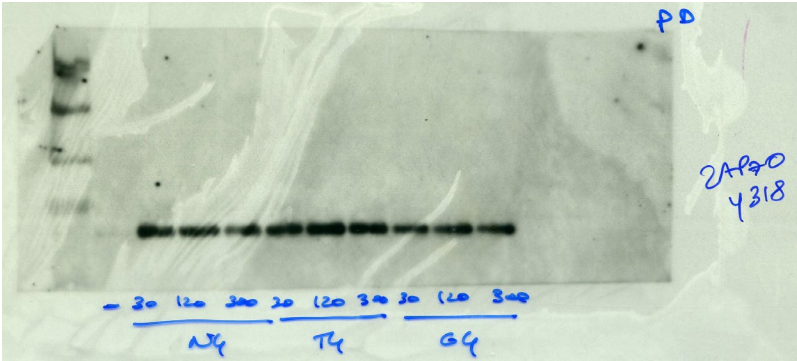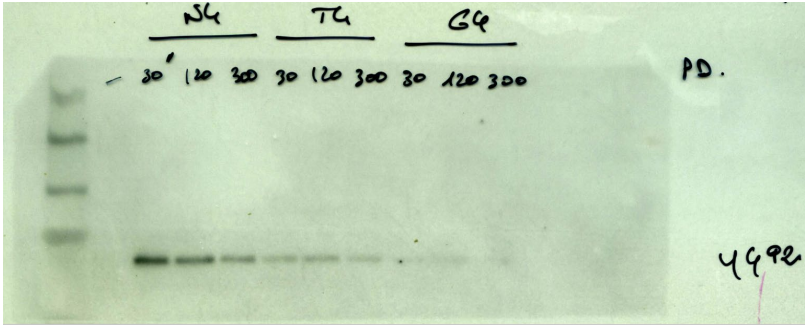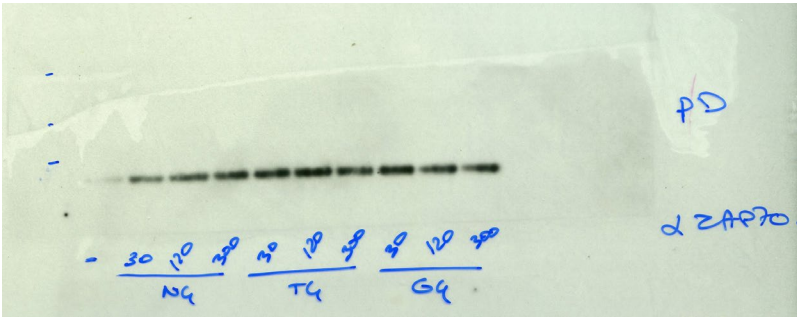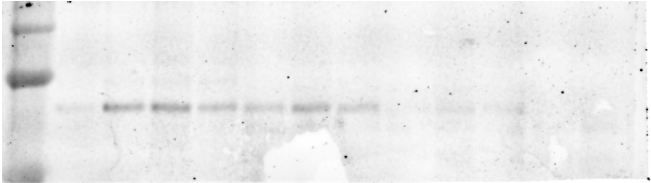

p-Y290-ZAP70

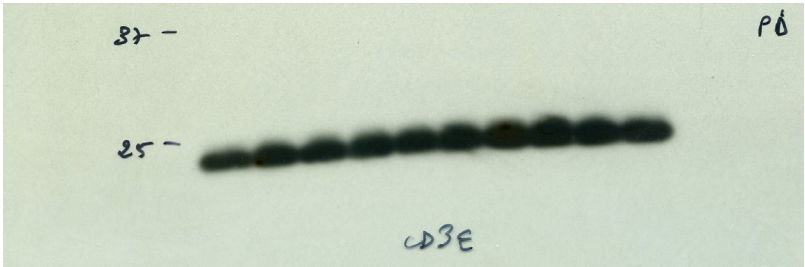

Supplement: Source Data Fig. 5 — Unprocessed western blots of Fig. 5. [file 41590_2022_1288_MOESM4_ESM.pdf]

Source Data-Figure 6b

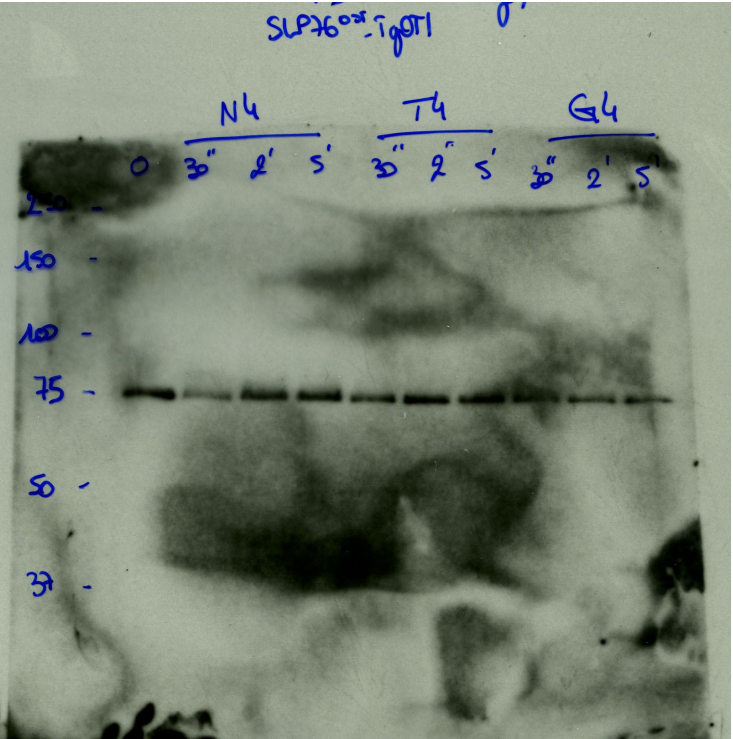

SLP76

Source Data-Figure 6c

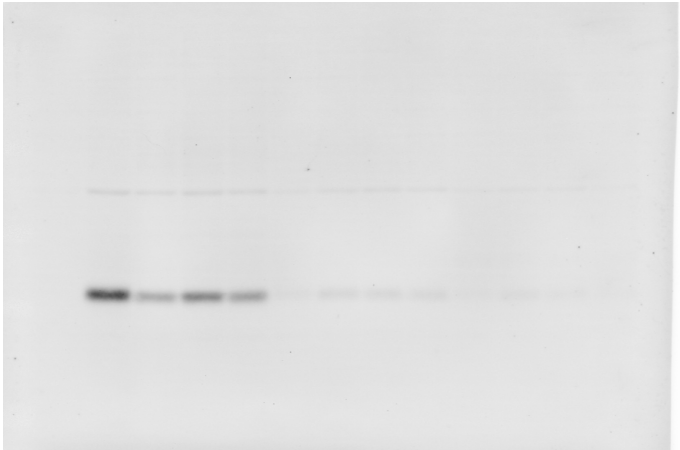

P-Y195 LAT

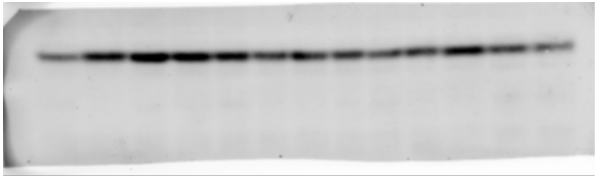

LAT

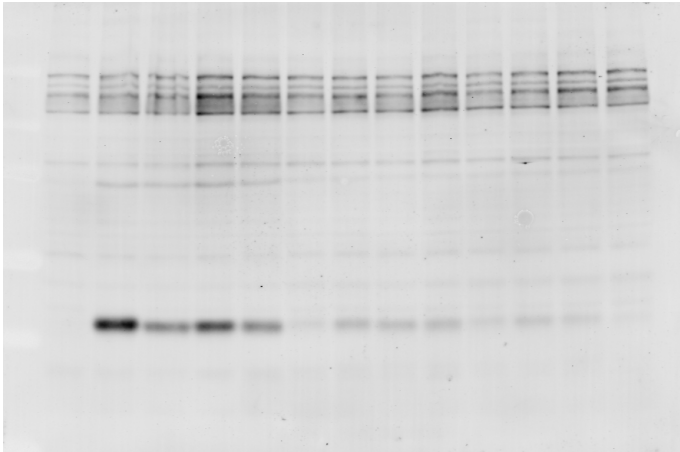

P-Y235 LAT

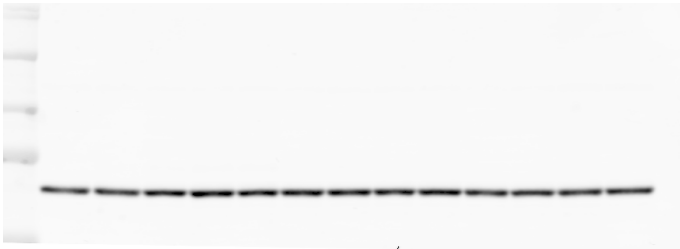

ZAP70

Supplement: Source Data Fig. 6 — Unprocessed western blots of Fig. 6. [file 41590_2022_1288_MOESM5_ESM.pdf]

Source Data-Extended Data Fig 5a

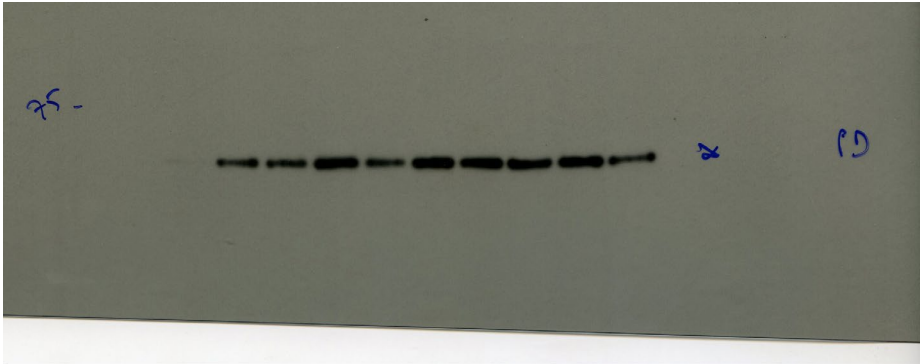

ZAP70

Source Data-Extended Data Fig 5b

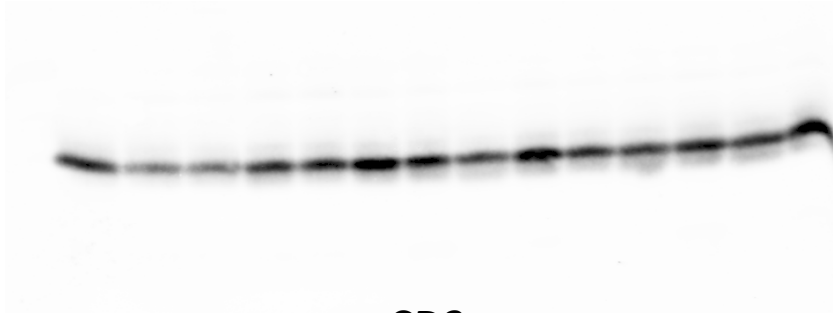

CD3z

Supplement: Source Data Extended Data Fig. 5 — Unprocessed western blots of Extended Data Fig. 5. [file 41590_2022_1288_MOESM7_ESM.pdf]

Source Data-Extended Data Fig 6b

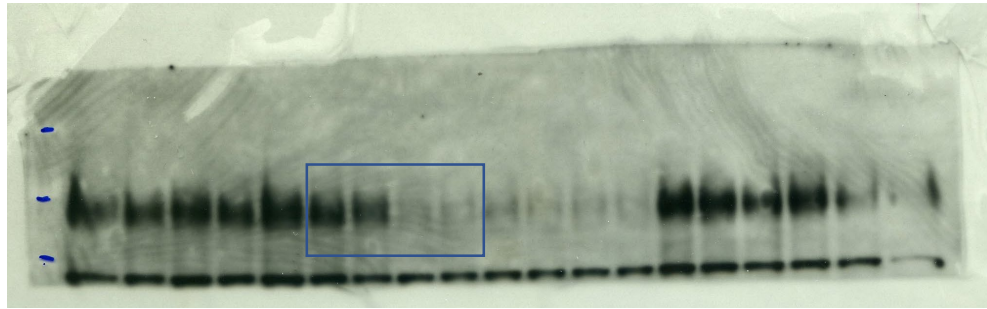

CD6

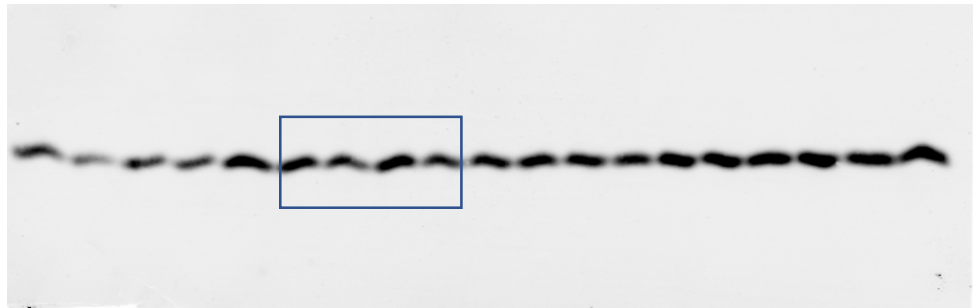

GAPDH

Source Data-Extended Data Fig 6c

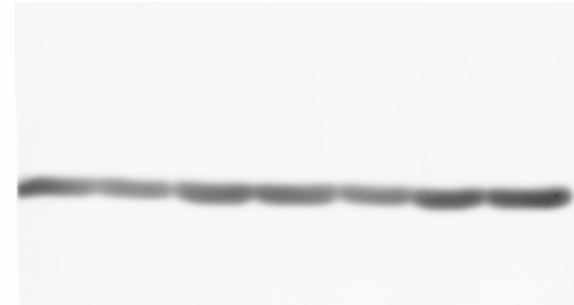

GAPDH

Supplement: Source Data Extended Data Fig. 6 — Unprocessed western blots of Extended Data Fig. 6. [file 41590_2022_1288_MOESM8_ESM.pdf]
