# Supplementary material for: Kinetic proofreading through the multi-step activation of the ZAP70 kinase underlies early T cell ligand discrimination
Source: Nat Immunol. 2022 Aug 31;23(9):1355–64. doi: 10.1038/s41590-022-01288-x (PMC9477740; doi:10.1038/s41590-022-01288-x)
Supplement: Source Data Extended Data Fig. 3 — Unprocessed western blots of Extended Data Fig. 3. [file 41590_2022_1288_MOESM6_ESM.pdf]

Source Data-Extended Data Fig 3e

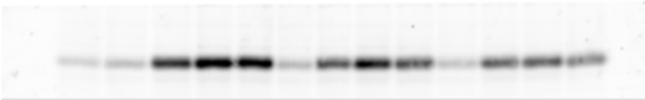

pS487 PCDCD4

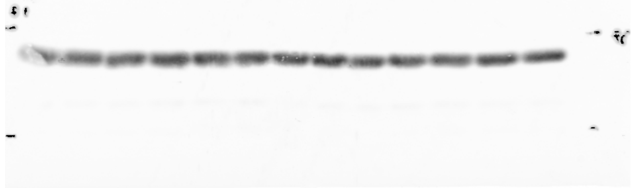

GAPDH

Source Data-Extended Data Fig 3g

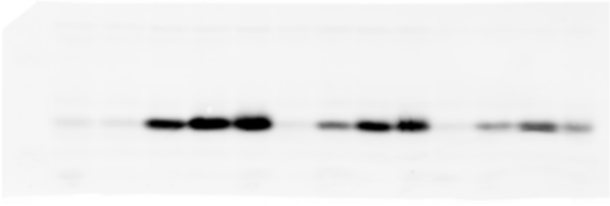

RPS6-pS235/236

Source Data-Extended Data Fig 3h

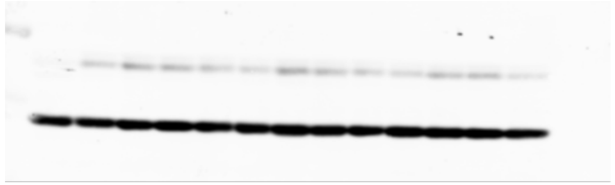

GAPDH

Source Data-Extended Data Fig 3j

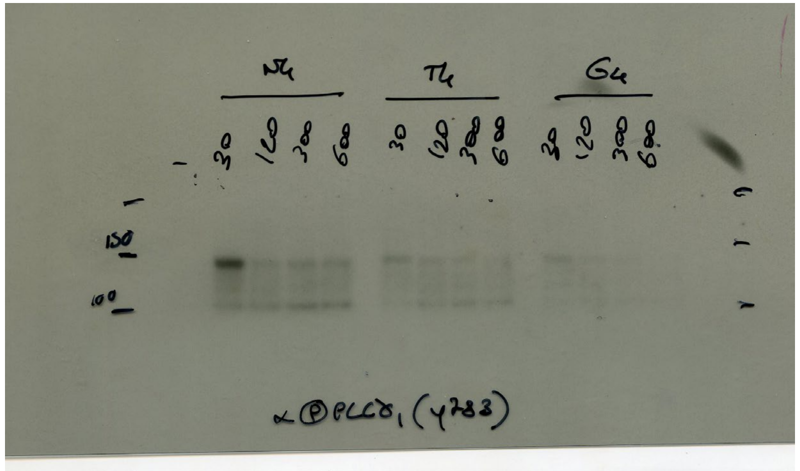

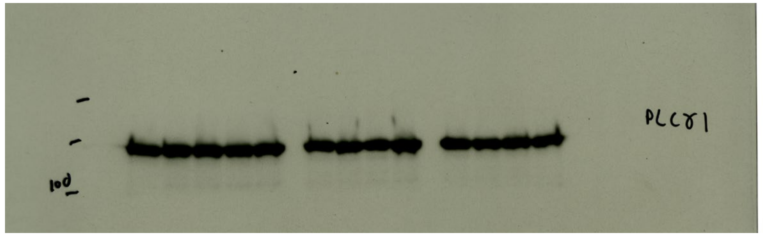

Source Data-Extended Data Fig 3f

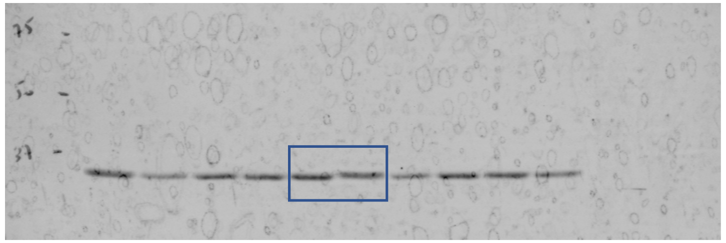

GAPDH
